# A State-of-the-Art Overview on (Epi)Genomics and Personalized Skin Rejuvenating Strategies

**DOI:** 10.3390/pharmaceutics17121585

**Published:** 2025-12-09

**Authors:** Roxana-Georgiana Tauser, Ioana-Mirela Vasincu, Andreea-Teodora Iacob, Maria Apotrosoaei, Bianca-Ștefania Profire, Florentina-Geanina Lupascu, Oana-Maria Chirliu, Lenuta Profire

**Affiliations:** 1 Department of Pharmaceutical and Therapeutical Chemistry, Faculty of Pharmacy, Grigore T. Popa University of Medicine and Pharmacy Iasi, 16 Universitatii Street, 700115 Iasi, Romania; roxana.tauser@umfiasi.ro (R.-G.T.); andreea.panzariu@umfiasi.ro (A.-T.I.); apotrosoaei.maria@umfiasi.ro (M.A.); florentina-geanina.lupascu@umfiasi.ro (F.-G.L.); oana-maria.ionescu@umfiasi.ro (O.-M.C.); lenuta.profire@umfiasi.ro (L.P.); 2 Department of Internal Medicine, Faculty of Medicine, Grigore T. Popa University of Medicine and Pharmacy Iasi, 16 Universitatii Street, 700115 Iasi, Romania; bianca-stefania.profire@umfiasi.ro

**Keywords:** photoageing, (epi)genomics, personalized skin rejuvenation, regenerative medicine, epigenetic drugs, stem-cell exosomes, sirtuins, microRNA, HOTAIR, antioxidants

## Abstract

This article aims to point out new perspectives opened by genomics and epigenomics in skin rejuvenation strategies which target the main hallmarks of the ageing. In this respect, this article presents a concise overview on: the clinical relevance of the most important clocks and biomarkers used in skin anti-ageing strategy evaluation, the fundamentals, the main illustrating examples preclinically and clinically tested, the critical insights on knowledge gaps and future research perspectives concerning the most relevant skin anti-ageing and rejuvenation strategies based on novel epigenomic and genomic acquisitions. Thus the review dedicates distinct sections to: senolytics and senomorphics targeting senescent skin cells and their senescent-associated phenotype; strategies targeting genomic instability and telomere attrition by stimulation of the deoxyribonucleic acid (DNA) repair enzymes and proteins essential for telomeres’ recovery and stability; regenerative medicine based on mesenchymal stem cells or cell-free products in order to restore skin-resided stem cells; genetically and chemically induced skin epigenetic partial reprogramming by using transcription factors or epigenetic small molecule agents, respectively; small molecule modulators of DNA methylases, histone deacetylases, telomerases, DNA repair enzymes or of sirtuins; modulators of micro ribonucleic acid (miRNA) and long-non-coding ribonucleic acid (HOTAIR’s modulators) assisted or not by CRISPR-gene editing technology (CRISPR: Clustered Regularly Interspaced Short Palindromic Repeats); modulators of the most relevant altered nutrient-sensing pathways in skin ageing; as well as antioxidants and nanozymes to address mitochondrial dysfunctions and oxidative stress. In addition, some approaches targeting skin inflammageing, altered skin proteostasis, (macro)autophagy and intercellular connections, or skin microbiome, are very briefly discussed. The review also offers a comparative analysis among the newer genomic/epigenomic-based skin anti-ageing strategies vs. classical skin rejuvenation treatments from various perspectives: efficacy, safety, mechanism of action, evidence level in preclinical and clinical data and regulatory status, price range, current limitations. In these regards, a concise overview on senolytic/senomorphic agents, topical nutrigenomic pathways’ modulators and DNA repair enzymes, epigenetic small molecules agents, microRNAs and HOTAIRS’s modulators, is illustrated in comparison to classical approaches such as tretinoin and peptide-based cosmeceuticals, topical serum with growth factors, intense pulsed light, laser and microneedling combinations, chemical peels, botulinum toxin injections, dermal fillers. Finally, the review emphasizes the future research directions in order to accelerate the clinical translation of the (epi)genomic-advanced knowledge towards personalization of the skin anti-ageing strategies by integration of individual genomic and epigenomic profiles to customize/tailor skin rejuvenation therapies.

## 1. Skin Ageing: Classification and Main Hallmarks

Skin ageing is a multifactorial process mediated by both intrinsic factors (such as time, genetics, hormones) and extrinsic environmental factors (i.e., lifestyle, nutrition, pollution, drugs, UV exposure) which accelerate the chronological (intrinsic/calendar) ageing [1,2]. Types of ageing including those referring to skin are presented in Table 1.

The main physiological and histological changes in aged skin phenotype might be described as follows: reduced elasticity and hydration; formation of wrinkles; sagging; itching; significant thinning and atrophy of the dermis and epidermis; altered barrier function; increased vulnerability to injury and disease; delayed wound healing; subcutaneous fat loss; uneven pigmentation; flattening of the epidermis–dermal connection; declining proliferative potential of the epidermal stem cells; collagen fragmentation and loss (approximately 1% yearly decline in collagen content in the dermis caused by parallel reduction in synthesis and increased degradation of existing collagen); disruption of the elastic fibre network; reduction in glycosaminoglycans and proteoglycans; impaired cell division of the melanocytes, of the cells in the germinative layer and those located in epidermis [2,10,11,12,13,14]. Similar genetic, biochemical and cellular disfunctions are involved in the visible signs of both chronologically aged and photoaged skin.

Intrinsic ageing and photoageing both share mechanisms like oxidative stress, senescence, and extracellular matrix (ECM) degradation, but photoageing accelerates these processes via UV-specific pathways involving stimulation of the matrix metalloproteases (MMP) activation and inflammation. Chronic localized inflammation in photoaged skin, with elevated cytokine expression (interleukin IL-6, tumour necrotic factor TNF α) and reactive oxygen species (ROS), contrasts with the low-grade, systemic inflammageing observed in chronological ageing; in addition, deoxyribonucleic acid (DNA) damage and cellular senescence accumulate more rapidly in UV-exposed skin areas [15]. Photoaged skin exhibits severe clinical signs (deep wrinkles, pigmentation, vascular changes) and histological alterations (solar elastosis, collagen fragmentation and degradation, faster ECM decline) that exceed those seen in chronological ageing [15,16,17]. Comparative insights on various features of the intrinsic aged and photoaged skin are illustrated in Table 2.

The main hallmarks of accelerated skin ageing are: (a) cellular senescence and the emergence of the Senescence-Associated Secretory Phenotype (SASP) associated with the accumulation of the senescent fibroblasts in the dermis, which are responsible for collagen degradation by the secreted matrix metalloproteases (MMP) and for secretion of pro-inflammatory factors; (b) genomic instability, due to the increased accumulation of DNA damage, mutations and genomic rearrangements; (c) telomere attrition (the gradual shortening of telomeres limiting the regeneration capacity); (d) epigenetic changes, like: DNA methylation, histone modifications, non-coding ribonucleic acid (RNA) regulation and heterochromatin-modifying gene expression patterns; (e) stem cell exhaustion, affecting tissue repair and renewal; (f) dysregulation of nutrient-sensing pathways, like mammalian target of rapamycin, (mTOR), insulin-like growth factor 1 (IGF-1) and IGF-1 signalling (IIS) pathway, AMP-activated protein kinase (AMPK, an energy-sensing enzyme), which alter metabolism and energy balance; (g) mitochondrial dysfunction, leading to impaired energy production and increased oxidative stress; (h) altered proteostasis, with the accumulation of misfolded proteins and Advanced Glycation End Products (AGEs), which is highly correlated to cellular senescence and plays a role in photoageing due to abnormal aggregation of glycated elastin fibres; (i) inflammageing; (j) altered intercellular communication and disconnectivity of the transcriptional networks; (k) microbiome disturbance; (l) compromised macro-autophagy [10,18,19,20,21,22].

Personalized and effective topical and systemic skin anti-ageing and rejuvenation strategies should target the above mentioned cellular, metabolic and (epi)genomic hallmarks, combined with minimally invasive procedures and nanotechnology, and are currently focused especially on: senotherapeutics (senolytics and senomorphics); regenerative medicine in order to restore skin-resided stem cells, based on mesenchymal stem cells or cell-free products (platelet-rich fibrin, autologous conditioned serum); epigenetic partial reprogramming; various epigenetic drugs (DNA methyltransferase inhibitors, histone deacetylase inhibitors, microRNAs, modulators of sirtuins and of long-non-coding RNA HOTAIR, telomeric repeat binding factor 2 TRF2); antioxidants (resveratrol, topical or oral use of various polyphenol-rich plants); pharmacological activation of autophagy; restoration of mitochondrial integrity essential for metabolism homeostasis [11,19,20,23,24,25].

The aim of this review is to concisely discuss: the clinical relevance of the most important clocks and biomarkers used in skin anti-ageing strategy evaluation, the fundamentals, the main illustrating examples preclinically and clinically tested, the critical insights on knowledge gaps and future research perspectives concerning the most relevant skin anti-ageing and rejuvenation strategies based on novel epigenomic and genomic acquisitions. Thus, the review dedicates distinct sections to senotherapeutics; stimulators of the DNA repair enzymes and of the proteins essential for telomeres’ recovery and stability; regenerative medicine based on stem-cells exosomes, genetically and chemically induced skin epigenetic reprogramming, modulators of microRNA and long-non-coding RNA (HOTAIR’s modulators) assisted or not by CRISPR-gene editing technology (CRISPR: Clustered Regularly Interspaced Short Palindromic Repeats), small molecule modulators of DNA methylases, histone acetylases, telomerases or of sirtuins, as well as modulators of the most relevant altered nutrient-sensing pathways and of mitochondrial dysfunctions and oxidative stress levels. The possibilities to positively influence skin inflammageing, proteostasis, (macro)autophagy and intercellular connections or skin microbiome, are also very briefly presented. Moreover, this review points out a comparative analysis among the newer genomic/epigenomic-based skin anti-ageing strategies vs. classical skin rejuvenation treatments from various perspectives: efficacy, safety, mechanism of action, price range, current limitations, evidence level in preclinical and clinical data and regulatory status. In these regards, a concise overview on senolytic/senomorphic agents, topical nutrigenomic pathways’ modulators and DNA repair enzymes, epigenetic small molecules agents, microRNAs and HOTAIRS’s modulators, assisted or not by CRISPR gene-editing technology, is illustrated in comparison to classical approaches such as tretinoin and peptide-based cosmeceuticals, topical serum with growth factors, intense pulsed light, laser and microneedling combinations, chemical peels, botulinum toxin injections, dermal fillers. Finally, this review emphasizes the future research directions in order to accelerate the clinical translation of the (epi)genomic-advanced knowledge towards personalization of the skin anti-ageing strategies by integration of individual genomic and epigenomic profiles to customize/tailor skin rejuvenation therapies.

## 2. Clocks and Biomarkers in Skin Anti-Ageing Strategy Evaluation: Description and Clinical Relevance

Chronological age is fixed and does not reflect skin health; biological age is more insightful for assessing rejuvenation [3]. The main biomarkers for estimating the biological age and assessment of anti-ageing interventions efficacy are integrated into epigenetic and non-epigenetic clocks, comprising DNA methylation-based biomarkers (epigenetic clock); analysis of key age-associated genes; cellular ageing hallmarks (SASP, altered macromolecules, genomic instability and shortened telomeres; senescence-associated heterochromatin foci SAHF) [26,27,28].

Among the skin-ageing biomarkers, DNA methylation-based (DNAm) epigenetic clocks, developed by regression models based on the increasing relationship with age of chosen groups of CpGs, are considered the gold standard in primary and accurate candidate metric at the molecular level and are highly reproducible and validated by most studies, both in ageing prediction and in skin rejuvenation research [29,30,31]. Such “epigenetic clocks” based on CpGs profile in skin fibroblasts, keratinocytes, endothelial cells, as well as in blood samples, would enable a better prediction and personalization of the skin rejuvenating topical and systemic senotherapeutics [29,32,33,34].

Based on a large-scale epigenome-wide association study (EWAS), altered DNA methylation patterns at CpG sites near *ECM* genes, such as elastin (*ELN*), lysyl oxidase (*LOX*), collagen type VIII alpha 1 chain (*COL8A1*), and matrix metallopeptidase 3 (*MMP3*) genes, have been associated with perceived/visibly aged skin [35]. These genes are interconnected within phenotypic/biological ageing of skin. The *ELN* gene encodes tropoelastin, an essential protein of the elastin fibres in the connective tissue. *LOX* encodes the enzyme responsible for crosslinking elastin and collagen fibres which strengthen *ECM* and play a key role in skin remodelling and wound healing. *COL8A1* contributes to collagen synthesis and thus slows down the ageing process, whilst *MMP3* catalyzes collagen degradation [34,36]. Boroni M. et al. have calculated skin-specific DNAm age based on the 2266 CpG sites within hundreds of cultured cells and human skin biopsies. Their model has enabled a highly accurate chronological age prediction, which is also sensitive to skin disorders, cell passage, or therapy with senotherapeutics. The tissue-specific DNAm age predictors are endowed with superior prediction power over the similar pan- or multi-tissue tests [36]. What remains to be further established is either the causal or the consequential relationship between DNAm profile and the skin ageing process/status. Initially regarded as a valuable tool to estimate chronological age at the molecular level, DNAm of biological samples has been recently reconsidered as a powerful tool to assess also the lifespan, longevity, the overall health status and mortality risk, due to its dependence both on time and additional factors, such as lifestyle and some genetic, inflammatory, metabolic and infectious disorders [34,35,36].

The epigenetic clocks are classified into generations, the first generation being mainly represented by Horvath clock, which is a universal clock detecting DNAm at 353 CpGs across tissues, without optimization for skin anti-ageing or short-term interventions [6]. Systemic epigenetic clocks (like GrimAge) are less useful for localized skin ageing evaluation but remain valuable in broader ageing-related drug development where skin is one of multiple targeted tissues [37]. The skin-optimized epigenetic clocks from second generation (e.g., Skin and Blood Clock, GrimAge, PhenoAge) and third generation (e.g., DunedinPACE, miRNA Skin Clock, Rayan Clock) provide measurable and sensitive biomarkers for skin-targeted interventions and they are most clinically valuable in dermatology and regenerative applications [4,5,6,26,27,28]. The Skin and Blood Clock is currently the most accurate skin-specific biological age tool, it provides the most clinically relevant and accurate measure of biological skin age, making it ideal for assessing the efficacy of cosmeceuticals, laser-based treatments, and cellular therapies targeting dermal rejuvenation; so, it is regarded as the benchmark for evaluating dermatological and regenerative therapies [6,26].

Pace-of-ageing clocks (e.g., DunedinPACE) enable monitoring of responsive treatment effects over short periods. DunedinPACE complements skin strategies by offering real-time insights into ageing velocity, especially in trials involving nicotinamide adenine dinucleotide NAD^+^ boosters or gene therapies; it also enables short-term tracking of intervention response [7]. Micro-ribonucleic acid (miRNA)-based Skin clocks show promise for non-invasive, patient-compliant diagnostics and testing, particularly in cosmetic dermatology [27]. Thus, emerging third generation clocks such as DunedinPACE and miRNA-based skin clocks offer novel value in the efficacy’s evaluation of skin rejuvenation strategies.

The last developed skin epigenetic clock by Rayan and collaborators represents a significant advancement by providing a tissue-specific biomarker that accurately reflects biological ageing and rejuvenation in human facial skin through DNA methylation profiling of epidermal and dermal fibroblasts. Its strength lies in sensitivity to reversible ageing changes following esthetic interventions, offering an objective measure for evaluating skin anti-ageing therapies. However, its reliance on invasive skin biopsies, limited commercial availability, and current validation primarily in lighter skin phototypes pose challenges for broader clinical application [26,28].

Mean Absolute Error (MAE) in skin epigenetic clocks quantifies the average difference between predicted skin biological age and the actual chronological age, serving as a key metric to evaluate the clock’s accuracy and reliability in measuring skin ageing. Among skin clocks and related ageing biomarkers, the Rayan Skin clock ranks highest with the lowest MAE (~3–4 years), outperforming earlier models like the Horvath Skin and Blood clock, thus reflecting its superior precision in assessing biological skin age. Within the MAE hierarchy, the Rayan clock is followed by the Horvath Skin and Blood clock (~5–7 years) and the blood-based clocks (like PhenoAge and GrimAge) which typically have higher MAEs when applied to skin (often >8 years) due to tissue specificity limitations. miRNA-based skin ageing clocks, still emerging, show moderate accuracy with MAEs around 8–10.9 years, but require further validation. This hierarchy highlights the importance of tissue-specific design for precise skin ageing measurement. Epigenetic biomarkers of (skin) ageing require further studies on inter-individual variability and also validation across diverse populations [26,28,34,35].

Besides epigenetic clock estimating cellular ageing by DNA methylation variations, non-epigenetic biomarkers, like telomeres’ length, senescence-associated proteins or inflammageing biomarkers, serve as mechanistic or histological adjuncts, not standalone age clocks, and they lack quantitative replication across patient cohorts, and the sensitivity and specificity needed for precision monitoring of clinical skin rejuvenation outcomes [8,9]. Among non-epigenetic senescent biomarkers might be considered: (a) p21, p53 (biomarkers of cell cycle arrest); (b) p16^INK4A^—its enhanced expression significantly correlates to chronological human epidermis ageing, older facial perception and to the age-changed elastin morphology in the dermal papilla (for instance, higher proportion of p16^INK4A^- positive melanocytes is associated with more facial wrinkles); (c) senescence-associated β-galactosidase (SAβGAL)—the mostly used histochemical biomarker in human dermal fibroblasts; (d) nuclear matrix protein lamin B1—a quantitative biomarker of skin senescence, decreasing with age; (e) acH4 (acetylated histone H4) and H4K20me1 (histone H4 monomethylated on lysine 20), which are altered histone biomarkers during skin senescence (correlated to delayed differentiation of keratinocytes and epidermal hyperplasia). Histones are DNA bound proteins with an essential role in chromatin organization, differentiation of epidermal stem cell niche into sebocytes and interfollicular epidermis, as well as in skin homeostasis [20,32,38,39]. In addition, the GlycanAge analysis measures changes in glycosylation patterns of immunoglobulins (IgG) which could trigger systemic chronic inflammageing and age-associated disorders [40,41].

Comparative insights on various epigenetic and non-epigenetic clocks/ biomarkers relevant to skin rejuvenation strategies’ evaluation are summarized in Table 3.

In conclusion, although photoageing remains a critical clinical concern with distinct histopathological markers which molecular clocks do not yet fully encompass, the integration of the biological age clocks, particularly epigenetic clocks, has revolutionized the evaluation of skin rejuvenation strategies, from topical interventions and energy-based devices to regenerative medicine and epigenetic drugs. A combined approach using validated epigenetic clocks with skin-specific calibration and complementary biomarkers offers the most powerful and practical framework for quantifying and validating the biological effects of anti-ageing interventions on the skin. These tools will be essential in personalizing dermatologic care, evaluating anti-ageing compounds, and guiding regenerative therapies in both clinical trials and esthetic medicine [26,28,34,35]. Precise biomarkers of ageing and rejuvenation for whole-body, at cellular and molecular levels, are prospecting the current advanced spatiotemporal multi-omics approaches, including transcriptomics, proteomics, metabolomics, gene signatures, and epigenomics, which can offer in-depth understanding of the complexities and relationships regarding molecular dynamics, cellular interactions and signalling pathways, inherent in ageing and regeneration, thus supporting new potential therapeutic innovations [20,30,43].

## 3. Senotherapeutics Targeting Senescent Skin Cells

### 3.1. Senolytics and Senomorphics—Mechanisms of Action

The senescent skin cells are characterized by: resistance to apoptosis; permanent loss of mitotic capacity; lower cell motility; over-activated focal adhesion kinases; activation of altered/inflammatory secretome (i.e., Senescence-Associated Secretory Phenotype, SASP); degradation of nuclear envelope lamina which anchors heterochromatin; remodelling of chromatin; reduced histone methylation (H3K9me3 and H3K27me3) with re-activation of silent DNA within highly condensed heterochromatin domains; the emergence of senescence-associated heterochromatin foci (SAHF); genomic instability and shortened telomeres; mitochondrial disturbances; morphological alterations. [10,14].

SASP might be triggered by multiple genome-related factors (DNA mutations, telomeres’ shortening, activated oncogenes, etc.) and consists of the release of pro-inflammatory cytokines (mainly interleukin IL-6 and IL-8), growth factors, proteases, bioactive lipids, metabolites, extracellular vesicles. All these secreted molecules will further promote chronic inflammation, degradation of ECM, avoidance of immune clearance of the senescent cells; moreover, it will enhance SASP secretion by an autocrine effect. The contribution of SASP to cellular senescence seems to be highly dependent on cell-type and stimuli [12,44]. Besides the altered secretome, skin cell senescence is also characterized by dysregulation of lipid metabolism with increased uptake and accumulation of lipids (presumably due to up-regulated p53 pathway and fatty acid synthase, which seems to be correlated to SASP), especially with increased levels of lysophosphatidylcholines and sphingolipids in human dermal fibroblasts [44,45]. In addition, in senescent cells there is an up-regulation of anti-apoptotic pathways, such as p53, Bcl-2 (B-cell lymphoma 2)*,* the heat shock protein 90 HSP90, nuclear factor kappa-light-chain-enhancer of activated B cells (inflammation pathway) (NF-kB), or nuclear transcription factor 2. For example, NF-κB is regarded as an essential transcription factor that controls the expression of multiple genes encoding SASP and pro-inflammatory cytokines and that is positively or negatively correlated with many other pathways involved in ageing, like mTOR, sirtuins, insulin/insulin-like growth factor 1 (IGF-1), DNA damage response. Therefore, the genetic down-regulation of NF-κB or its pharmacological inhibition by senomorphics have diminished cell senescence in mouse models. For instance, the compound SR12343 is an NF-κB inhibitor that has reduced in vivo SASP factors in senescent fibroblast cells and has prolonged lifespan in both chronologically aged and photoaged mice [10,12,44].

### 3.2. Main Senotherapeutic Agents Preclinically and Clinically Tested

Senotherapeutics can be divided in two classes: senolytics which selectively eliminate senescent cells by re-activating their apoptotic pathways, and senomorphics which mitigate the SASP without killing senescent cells; the latter are regarded as safer and more effective than senolytics [12,44,46]. The differentiation between two classes seems difficult in vivo and dependent on cell type and agent’s concentration [44].

A comparative analysis regarding the main outcomes and limitations of the most important senomorphic and senolytic agents tested for skin rejuvenation is presented in Table 4. In mice transplanted with human aged skin xenograft and treated for 30 days with the oral senolytic cocktail composed of dasatinib (a tyrosine kinase inhibitor) and the flavonoid quercetin (D + Q), the following results were reported: a substantial decrease in SASP factors (IL-6, MMP-1, MMP-3) and an enhanced dermal collagen proportion, in the absence of noticeable inflammatory reaction or side effects [47]. Mohammad IS et al. has found that a single dose of D + Q was able to induce temporary chromatin changes in young vascular smooth muscle cells and durable anti-senescent effects in senescent cells, the single dose being more selective than triple dosing; however, the off-target effects, especially on healthy cells, are not addressed [48]. In a clinical trial, after 3 days of oral administration, the senolytic cocktail D + Q lowered the expression of some biological age biomarkers, such as p16 and p21 in epidermis, as well as p16, p21, SAβGAL and SASP factors in adipose tissues; these effects were maintained even 11 days post-therapy [12,49]. To date, clinical trials have not assessed D + Q effects on visible or structural skin parameters (e.g., elasticity, wrinkle count), the potential off-target effects (dasatinib may temporarily induce senescence-like features in healthy cells), long-term toxicity (dasatinib might cause pleural effusion, cytopenias and immunosuppression); in addition, current clinical studies have open-label designs, short intervention duration (most were over 3–7 days, with only one study lasting 6 months), and enrolled small cohorts (on average 20 participants) without placebo controls [47,50].

The senolytic agent ABT737 is a specific inhibitor of Bcl-2 anti-apoptotic pathway and was able to induce ~65% cell death and to reduce the expression levels of SAβGAL, p16, p21, besides to an increased epidermal concentrations of the pro-apoptotic protein cleaved caspase-3 in cultures of human and mouse senescent skin fibroblasts with DNA mutations. Moreover, its derivative ABT263 (navitoclax), applied topically to mice, reduced the senescence biomarker p16^INK4A^, enhanced collagen network and dermal thickness, while attenuating hyperpigmentation, by selective elimination of fibroblasts in a murine model of senile lentigo [51,52]. The flavonoid fisetin is another promising senolytic that reduced MMPs and transepidermal dehydration in a mouse model of skin photoageing [33,53,54].

Rapamycin (sirolimus), an immunosuppressive drug, acts also as senomorphic by inhibition of mTOR and NF-κB, suppressing SASP factors. In topical administration, rapamycin reduced wrinkles, decreased the p16^INK4A^ biomarker of skin cells’ senescence to subjects over 40 years old with photoageing and loss of dermal volume [55,56,57]. As senomorphics, metformin, apigenin and kaempferol significantly decrease SASP in senescent fibroblasts [58,59]. The first-choice biguanide in type 2 diabetes, metformin suppresses SASP expression and SAβGAL activity in different senescent cell types, including human diploid fibroblasts. Furthermore, metformin modulates many of the interconnected pathways of biological ageing by: activation of AMP-activated protein kinase (AMPK) and sirtuin SIRT1; down-regulation of insulin/IGF-1 and mTOR; stimulation of DNA repair mechanisms and mitochondrial functions; reduction in oxidative damage, genome instability and telomeres’ shortening; stimulation of macroautophagy and proteostasis; reduction in epigenetic histone alterations. Metformin expands the lifespan in mouse models and has entered in TAME (Targeting Ageing by Metformin) clinical trial [33,60,61]. Some statins (atorvastatin, pravastatin, pitavastatin) inhibit oxidative stress-induced endothelial senescence and up-regulate endothelial nitric oxide synthase and SIRT1; simvastatin acts as senomorphic by decreasing SASP expression in senescent human fibroblasts [11,22,52]. Inhibitors of p38MAPK pathways such as UR13756 and BIRB796 have been very efficient in blocking the SASP, since p38MAPK is activated by various senescent-inducing factors and is also greatly involved in promoting cellular senescence [19,33].

The senotherapeutic peptide OS-01 (also known as Pep 14), the active ingredient in OneSkin’s topical products which have been patented since April 2025, acts both by preventing cells from entering into the senescent state and as senolytic against the existent senescent cells, in a similar way as topical rapamycin [62,63]. In randomized clinical trials (RCT), OS-01 has thickened the skin barrier, enhanced skin radiance and texture, and has also diminished the wrinkles’ depth. Moreover, in the OS-01-topically treated female volunteers group, there were recorded a significant decrease in the blood concentrations of the pro-inflammatory cytokine IL-8, as well as a reduced progression rate in biological age (measured by GlycanAge analysis) [64]. Clinical trials for the pipeline senotherapeutic peptide OS-01 have also underscore the skin’s essential role within both the systemic ageing network and the chronic inflammatory disorders, thus emphasizing the importance of integrating the skin-focused antiageing strategies within other pro-longevity ones [64,65].

BPTES, bis-2-(5-phenylacetamido-1,3,4-thiadiazol-2-yl)ethyl sulphide, is a selective inhibitor of glutaminase-1 (GLS1), enzyme that plays a critical role in survival of human senescent cells; inhibition of GLS1 could remove senescent cells and stimulate skin repair mechanisms [66]. In an interesting mouse/human chimeric model (skin grafts of senescence-induced human dermal fibroblasts collected from male volunteers which were subcutaneously transplanted to nude mice), BPTES, given as intraperitoneal (i.p.) injections, has demonstrated a significant and selective senolytic activity against aged human dermal fibroblasts, which was maintained up to 30 days post-therapy. BPTES has increased collagen’s density in the dermis, while it has reduced SASP biomarkers (SAβGal, p16, p21, IL-1β, IL-6, IL-8, MMP-1, MMP-3, MMP-9). Since glutaminase-1 and glutaminolytic pathways are also shared by the activation of immune T cells in response to cancer cells proliferation, their inhibition by BPTES might be associated with the risk of long-term carcinogenesis. Other limitations of this study are also presented in Table 4 [67,68].

FOXO4-DRI peptide (FOXO4-*D*-retro-inverso-isoform peptide) has selectively down-regulated p53-serine15 phosphorylation (p53-pS15) responsible for apoptosis resistance in keloid fibroblasts and has also promoted p53-pS15 translocation from the nucleus to the cytoplasm. Its promising anti-ageing effects and limitations into clinical translation are also depicted in Table 4 [69,70]. Other clinical trials are investigating the anti-senescence activity of procyanidin C1 (PCC1) and its senolytic complex PCC1 + Cellumiva™ (procyanidin C1 + pterostilbene + spermidine), as well as of cycloastragenol (CAG), 25-hydroxycholesterol, nordihydroguaiaretic acid (NDGA), rutin, *Silybum marianum* extract, urolithin A and ergothioneine, which are summarized also in Table 4 [60,71,72,73,74,75,76,77,78,79,80,81].

**Table 4:** Comparative analysis of the main senomorphic and senolytic agents preclinically and clinically tested for skin rejuvenation. (the arrow ↓ means decrease; the arrow ↑ means increase).

Substance	Model and Dose Regimen	Mechanism/ Modified Biomarkers	Main Results and Limitations	Ref.
SENOLYTICS				
Dasatinib + Quercetin (D + Q)	Mice model; oral 5 mg/kg D + 50 mg/kg Q, intermittent dosing;	↓ p16, p21 ↓ SAβGAL ↓ SASP Bcl-2 inhibition	Attenuated fibrosis, cognitive decline, osteoarthritis, diabetic complications; Limited administration period, small size cohorts; no data on visible or structural skin parameters; no long-term safety data (especially on healthy cells);	[12,47,49]
Navitoclax (ABT263)	Preclinical/animal studies; early-stage topical formulation development: topical application of ABT-263 (Navitoclax) in aged mice; 5 day treatment; Murine models of lung fibrosis, osteoarthritis; 50 mg/kg/day for 2 weeks;	Bcl-xL/Bcl-2 inhibitor; ↓ senescent cell viability; ↓ p16^INK4A^	Improved dermal thickness and collagen organization; reduction in skin senescence markers; improved wound healing in aged mice; improved tissue function; Toxicity concerns; human topical safety/efficacy not established; thrombocytopenia as adverse effect in systemic administration;	[51,52,72]
BPTES	Mouse/human chimeric model (skin grafts of senescent human dermal fibroblasts were subcutaneously transplanted to nude mice); mice treated with BPTES or vehicle, intraperitoneal, for 30 days	↓ SAβGal; ↓ p16, p21; ↓ IL-1β, IL-6, IL-8; ↓ MMP-1, MMP-3 and MMP-9	Selective senolytic effects on aged dermal fibroblasts, sustained 1 month post-therapy; increased collagen density; increased cell proliferation in the dermis; decreased SASP. Limitations: small sample size; only male human skin grafts collected; unknown effects on the surface properties of human skin; risk of suppression of the skin T lymphocytes’ proliferation and risk of carcinogenesis	[66,67,68]
FOXO4-DRI peptide (FOXO4-*D*-retro- inverso-isoform peptide)	Preclinical animal studies; Clinical trial: 10 keloid skin samples (females); 7 normal skin samples (female participants)	Peptide-induced senescent cell apoptosis: disruption of FOXO4–p53 interaction	Mechanistic insights into FOXO4-DRI: down-regulation of p53-serine15 phosphorylation (p53-pS15); p53-pS15 translocation into cytoplasm; selective agents to induce apoptosis of senescent fibroblasts in both keloid fibroblast and organ culture senescent models. Improved skin regeneration and reduced ageing biomarkers; rejuvenate epidermal stem cell function, leading to improved skin barrier integrity and repair capacity; Delivery challenges; small cohorts	[69,70]
Fisetin	C57BL/6 mice; 100 mg/kg/day, 1 week on/1 week off	↓ p16^INK4A^; Bcl-2 family inhibition	Improved vascular endothelial function and arterial stiffness	[54]
Cycloastragenol (CAG)	Aged mice; oral, 50 mg/kg/day for 2 weeks	↓ Bcl-2, PI3K/AKT/mTOR axis inhibition	Selective senescent cell clearance; improved cardiac and muscle function	[75]
25-Hydroxycholesterol	Aged mice; i.p., 50 mg/kg/day 5 consecutive days	↓ p16^INK4A^, ↓ IL-6, ↓ TNF-α	Reduced arterial stiffness and improved vascular reactivity	[76]
SENOMORPHICS				
Rapamycin/ sirolimus	Skin explants and 3D models; Human subjects, photoaged skin: topical; Mice model: intermittent or lifelong oral dosing (e.g., rapamycin 14 ppm in diet);	Inhibition of mTOR, NF-κB and SASP; ↓ p16^INK4A^ ↓ IL-6, IL-8 in plasma; ↓ MMP-1	Reduced wrinkles; improved ECM remodelling; extended lifespan, improved cardiac and cognitive function (mice model); Small cohort; reduced administration period	[55,56,57,62,63]
Metformin	HUVEC cells in vitro (0.5–2 mM); aged mice 50 mg/kg daily	↑ AMPK, ↓ NF-κB, ↓ ROS	Reduced SASP, improved endothelial function	[60,61]
Nordihydroguaiaretic acid (NDGA)	C57BL/6 male mice: 15 mg/kg/day oral	LOX inhibition, ↑ PPARα, ↑ AMPK	~8–10% lifespan extension, improved metabolic parameters	[81]
Rutin	Aged mice; 50 mg/kg/day oral	Inhibits ATM–HIF1α–TRAF6 axis; ↓ IL-6	Reduced vascular inflammation, enhanced chemotherapy efficacy	[77]
SENOMORPHIC AND SENOLYTIC (DUAL ACTION)				
OS-01 (Pep 14)	RCT	↓ SASP ↓ IL-8 ↓ glycated IgG	Thickened the skin barrier, enhanced skin radiance/texture, diminished the wrinkles’ depth. Limited cohorts	[64,65]
Procyanidin C1 (PCC1)	ageing-related skin-fibrosis, murine models	inhibition of epidermal growth factor receptor (EGFR) phosphorylation and suppression of multiple downstream signalling cascades (ERK/MAPK, AKT/mTOR and TGFβ/SMAD pathways)	Significant anti-fibrotic skin effects: reduced epidermal hyperplasia and thickness; reduced abnormal collagen deposition; restored the collagen I/III ratio human translation needs validation	[71,72,73]
PCC1 + Cellumiva™ (senolytic complex = procyanidin C1 + pterostilbene + spermidine)	Open-label RCT on 75 female healthy volunteers, aged 45–65; oral dietary supplement; once daily; 12 weeks	imaging technologies; feedback questionnaires	Good effects on skin barrier function and texture/radiance, while diminishing wrinkles; Limited safety data	[73,80]

### 3.3. Critical Insights on Senotherapeutics as Skin Rejuvenating Strategy

Senolytics and senomorphics can mitigate chronic senescence in aged skin by removal or modulation of senescent cells via targeting senescence pathways and can also rejuvenate tissue microenvironment, but they can be associated with the risk of excessive, unintended normal tissue clearance or inflammation [44,50,51,59]. Although more than 20 senotherapeutics are now in clinical trials, it remains to further investigate their efficacy’s dependency on the cellular type, concentration and stress factors (like for aspirin’s effects on senescence), thus guiding the choice of these agents in specific age-related skin diseases [10,19,82]. Moreover, it remains to elucidate: (a) the adverse effects of senotherapeutics on various proliferating or quiescent cell-types which share critical pathways with those in targeted senescent cells; (b) their dysregulatory potential of other critical cellular processes, as well as (c) their effects on many ageing biomarkers, in order to more precisely elucidate their mechanisms of action and to establish the best choice between senolytics and senomorphics (given the role of senescent cells in wound healing, tissue regeneration, cancer prevention) [12]. Synergistic effects of senotherapeutics with existing treatments (such as retinoids or antioxidants) might enhance the efficacy on skin rejuvenation but require future evidence-proof human trials [83]. Current limitations and future research directions on senotherapeutics as skin rejuvenators are depicted in Table 5.

## 4. Skin Anti-Ageing Strategies Targeting Genomic Instability and Telomere Attrition

Nuclear genome instability is mainly caused by the accumulation of DNA mutations and by the decline in the efficiency of the DNA damage repair (DDR) mechanisms. Nuclear genome instability is significanty correlated to an accelerated progression of age-related changes, as noticed also in progeroid Werner and Cockayne syndromes [84,85]. Telomeres are repetitive DNA sequences acting as protective caps at the extremities of linear eukaryotic chromosomes with a crucial role in genomic stability. During ageing and consecutive of multiple cell divisions, telomeres become progressively shorter; beyond a critical length of the telomeres, the cell cycle will be irreversibly stopped, thus triggering cell apoptosis. The parallel processes of telomeres’ shortening and of cell senescence, respectively, represent an essential hallmark of ageing called “life clock” [34,86]. Besides genomic instability, telomere attrition is viewed as a main contributor to fibroblast senescence in aged skin and disease-related ageing skin. Excessive telomere attrition in the progenitor cells of the highly proliferative tissues (haematopoietic system, gastrointestinal tract, skin) ultimately triggers DDR such as cell cycle arrest, apoptosis, differentiation disorders and senescence, while in hypoproliferative tissues (heart, brain and liver) oxidative stress might further increase telomere sequence damage and attrition [87]. DDR signalling pathways are activated in response to few critically short telomeres and comprise the following steps: the overexpression of p53 and p21 (cell cycle inhibitory biomarkers); the altered secretome SASP, which modify the ECM composition and propagates the senescent phenotype to surrounding cells; finally, systemic chronic inflammation [88,89].

The protective approaches to overcome telomere attrition tested so far could be illustrated by:

- stimulation of reverse transcriptase telomerase, an enzyme responsible for biosynthesis of new telomeric DNA based on RNA template in highly proliferative skin cells like stem cells, using for instance as telomerase activator the compound TA-65 [8,42]. Moreover, liposomes with xenogenic DNA repair enzymes like photolyase isolated from microalgae *Anacystis nidulans* and T4 endonuclease from *Micrococcus luteus* have proven efficient due to a significant decrease in telomere shortening rates [11,33]; as well as liposomes with 8-oxoguanine glycosylase photolyase (OGG1) which have demonstrated an essential role in reducing the biomarker 8-oxo-7,8-dihydro-2′-deoxyguanosine of oxidative DNA damage and mitochondrial dysfunctions [58,84];
- recovering of the multi-protein shelterin complex that is essential for stabilization of chromosomes and for their protection against being detected as double-stranded breaks. The TRF2 protein of shelterin complex is involved in telomere capping and DDR inhibition; TRF2 deficiency triggers the activation of p53 signalling pathway and cellular apoptosis. In this regard, topical application of TRF2 might have a protective role in telomeric DNA [58,85,87];
- nicotinamide adenine dinucleotide NAD^+^ boosters (such as NAD^+^ precursors: nicotinamide riboside and nicotinamide mononucleotide), which, like telomerase activators (e.g., compound TA-65), support DNA integrity and cellular longevity, help slow DNA damage accumulation; however, direct evidence in human skin is limited and their effects may be systemic [59].

## 5. Regenerative Medicine Targeting Skin Stem Cell Exhaustion: Fundamentals and Clinical Progress

Mesenchimal stem cells (MSCs) have an essential role in tissue repair and homeostasis, due to their multipotency and self-renewal ability. Aged skin is marked by stem cells’ pool declining, correlated with skin atrophy, fragility and hyperpigmentation [86,90]. Regenerative therapeutic approaches in skin rejuvenation are designed upon the current understanding of the main mechanisms and molecules involved in the physiological regeneration of skin. Using either endogenous stem cells, such as adipose-derived mesenchymal stem cells (AD-MSCs), amniotic membrane stem cells (AMSCs), human umbilical cord mesenchymal stem cells (hUC-MSCs), tissue-induced pluripotent stem cells (iPSCs), or extracellular vesicles/exosomes derived from endogenous MSCs, the following beneficial anti-ageing effects were reported: stimulation of mitosis, proliferation and differentiation, especially of human dermal fibroblasts; up-regulation of local immunological defence mechanisms; release of the angiogenesis-modulatory cytokines, antibacterial peptides and anti-inflammatory molecules; release of the growth factors and cytokines essential for skin trophicity, tissue re-epithelization, wound healing, biosynthesis of elastin and collagen fibres in the ECM; inhibition of proteins’ glycation; inhibition of oxidative stress [38,45,84,91]. The stem cells have the advantage of being easily obtained and abundant. Other advantages, especially of AMSCs, rely on: multipotency, low immunogenicity, facil isolation from the placenta, unapplicable ethical issues like those imposed to embryonic stem cells [18,92].

Regenerative medicine-based skin therapies used alone or in combination to conventional treatments might offer more promising, efficient and safe solutions in facial rejuvenation, in comparison to conventional approaches (such as cosmeceuticals, microneedle, dermal fillers, injectables, fractional laser) [12,92,93]. For instance, topical application of AMSC with vitamin E to photoaged human subjects have significantly decreased wrinkles, ultraviolet spots and pores. Injected into the dermis, ADSCs have improved skin vascularization, hydration and density. In topical use, hUC-MSCs-conditioned media associated with microneedling significantly improved skin brightness and texture in comparison to microneedling alone [18,93].

On the other hand, as an alternative to stem cell-based skin rejuvenation, regenerative cell-free approaches might use: (1) topical platelet concentrates, such as: platelet-rich plasma (PRP), platelet-rich fibrin (PRF), and injectable platelet-rich fibrin (i-PRF); and (2) blood cell secretome (BCS). PRP, an autologous and highly concentrated preparation of platelets, is extensively applied in skin anti-ageing because it has shown to boost: (a) skin rejuvenation through the great proportion of growth factors (i.e., platelet-derived growth factor PDGF, transforming growth factor TGF, vascular endothelial growth factor VEGF, and IGF-1), which are essential for fibroblasts’ proliferation and for biosynthesis of the dermal collagen and elastine fibres; (b) thickening of epidermal layers and of dermal–epidermal junction; (c) wound healing; (d) recovery of oxidative homeostasis [22,38,53]. Regarded as second- and third-generation platelet concentrates, PRF and i-PRF have been so far applied in limited clinical trials to establish their effectiveness in skin rejuvenation [85]. BCS, also called autologous conditioned serum (ACS), is obtained by the enrichment of blood serum with anti-inflammatory cytokines, growth factors, lipid mediators, and exosomes. In a clinical study, topical application during 12–24 weeks of BCS significantly enhanced skin hydration and firmness [31,91].

In preclinical and clinical studies, stem-cells-derived exosomes (extracellular vesicles, EVs) as rejuvenation therapies have demonstrated the ability to modulate the expression of some longevity-associated genes (i.e., *Klotho*, *FOXO3*, *FGF23*) or collagen-related genes (*COL1A1*); to stimulate ECM remodelling, to increase collagen synthesis and elastin fibre density, thus enhancing dermal thickness; to modulate inflammation and to promote angiogenesis via PI3K/AKT, Notch pathways; to improve skin hydration, elasticity, tone and pigmentation (usually within 6–12 weeks) and to reduce wrinkles. Adipose-derived mesenchymal stem cell (AD-MSC) secretome or other stem-cells-derived exosomes investigated for facial skin rejuvenation have gained more effective transdermal delivery when coupled to microneedling, intradermal injections or fractional laser; moreover, these adjunctive methods have enabled an increased collagen production, ECM remodelling and more profound biological impact than topical-only applications. The choice among these delivery methods is dependent on the side effects’ risks, patient preferences and costs. Stem-cells-derived exosomes are well-tolerated, with minimal erythema or petechiae from microneedling or laser. For instance, microneedling is associated with fewer reported adverse reactions (transient erythema, edema, pain and discomfort for several hours post-treatment), is cheaper and more accessible than fractional laser [94,95,96]. All these above-mentioned efficacy and tolerability data are confirmed and validated mainly from a cosmetic-dermatologic perspective. In addition, most clinical trials enrolled small cohorts (*n* < 60 participants), with short-term follow-up (≤12 weeks) and lacked robust controls (e.g., placebo, double-blind, randomized large size arms), therefore restricting their generalizability and statistical power of results [94,95,97]. Moreover, few clinical trials directly assess biomarkers specific for epigenetic age (e.g., DNA methylation), therefore the epigenetic profiling still remains an unmet opportunity in human skin rejuvenation trials [95,97].

The main aspects of the clinical trials on stem-cells derived exosomes (EV) as skin anti-ageing strategy are described in Table 6, comprising study design, the principal results and limitations. analyzing the preliminary results of the clinical interventions for stem-cell-based exosomes used alone or in combination with traditional methods, we can conclude that stem cell-derived EVs are the most clinically advanced biologic interventions in skin anti-ageing and rejuvenation, demonstrating improvements in skin texture, pigmentation and acne-scars’ healing. Their regenerative and therapeutic potential in skin anti-ageing or skin repair is worthing further investigations in larger-scale RCTs, with better standardization of the study’s design and optimized formulations. Current obstacles in regenerative therapies translation into clinic are related to tissue sources, isolation technology, production control over batches, limited large clinical trials for efficacy and potential adverse reactions, lack of standardized protocols, as well as ethical legislation [92,98,99,100,101,102,103,104,105,106,107,108].

## 6. Regenerative Medicine Focused on Epigenetic Reprogramming and Epigenetic Drugs

### 6.1. Fundamentals of Epigenetic Reprogramming as (Skin) Rejuvenation Strategy

Progressive and persistent epigenetic alterations are regarded as primary drivers of the accelerated skin ageing process and somatic cells heterogeneity. Deciphering the dynamics and molecular mechanisms of epigenetic modifications during ageing is essential for therapeutic interventions’ design in skin rejuvenation and age-related diseases treatment, such as transient in vivo reprogramming [91,109]. Research on various models and species of ageing-related phenotypes and age-related diseases from yeast to human cells have identified the main age-related epigenetic alterations:

✓ changes in DNA methylation pattern at cytosine residues, which are highly tissue-and age-specific;
✓ post-translational covalent alterations of histones (i.e., methylation, acetylation, phosphorylation), decreased proportion of core histones and the incorporation of non-canonical histones;
✓ reduced global heterochromatin and heterochromatin structural modifications with accumulation of senescence-associated heterochromatin foci (SAHF);
✓ dysregulation of non-coding RNA’s expression pattern (ncRNA, i.e., microRNAs miRNAs, long non-coding RNAs lncRNA, and circular RNAs circRNA), which is correlated to alterations of the expression of genes involved in inflammation and oxidative stress-mediated cellular senescence [85,89,109].

Epigenetic reprogramming is one of the most promising emerging in vivo and in vitro skin-rejuvenation strategies able to reverse the transcriptome of the senescent cells in aged skin, by concomitantly ameliorating multiple skin-ageing hallmarks: telomere size, gene expression profiles, oxidative stress levels, mitochondrial morphology and metabolism, and nuclear envelope integrity. Epigenetic reprogramming-induced rejuvenation can be mediated either by transcription factors (genetically induced reprogramming) or by small molecules (chemically induced reprogramming, for instance by DNA methyltransferase inhibitors and histone deacetylase inhibitors) [84,86,87].

Having a huge potential in regenerative medicine, epigenetic reprogramming-induced rejuvenation might be complete or partial, although for anti-ageing strategies the partial reprogramming is much more adequate. Complete reprogramming converts any type of somatic cells into iPSCs, which have self-renewal ability and the potential to redifferentiate into fully rejuvenated various cell types. These features are mediated by complex and interconnected networks of transcription factors, signalling molecules and genes expression profiles, which make the process of ageing strongly linked to cellular differentiation [88,89]. Since complete dedifferentiation is also a common oncogenetic process, partial epigenetic rejuvenation aims to avoid the risk of tumorigenesis and to separate the rejuvenative properties and the safe age-reverse of reprogramming from full dedifferentiation, thus preserving the original cell phenotype, instead of regaining pluripotency [51,93,110,111].

### 6.2. Genetically Reprogramming-Induced (Skin) Rejuvenation Strategies

Genetically reprogramming-induced rejuvenation strategies currently involve the transient overexpression of four transcription factors called Yamanaka factors or OSKM factors (4F: OCT4, SOX2, KLF4 and c-Myc) able to trigger dedifferentiation in any type of somatic cell and reverse their ageing clocks, while preserving the cellular identity. In order to avoid the tissue-specific dysplasias reported for continuous expression of the OSKM/4F during 4–7 days, as well as the high risk of tumorigenesis (teratomas in mammalians) reported for in vivo 4F expression longer than 8 days, the epigenetic rejuvenation is applied as transient and shorter 4F induction cycles [45,88]. In addition to multiple short cycles of OSKM expression, there is a safe timeframe of reprogramming-induced rejuvenation and also a critical recovery period after treatment (since many biomarkers of transcriptome reversal occurred weeks later), not to mention that reprogrammed cells secrete soluble factors able to rejuvenate non-reprogrammed cells [38,84,85].

In the physiologically ageing mice, a single period or short cycles of OSKM expression have induced, in various tissues (including skin) and at body level, reversal of epigenetic clock, transcriptomic and metabolic effects, such as: DNA methylation changes, as well as lower expression of genes mediating inflammation, senescence and stress response pathways [38,112]. For instance, ubiquitous multiple transient cycles of 4F expression (2 days of expression followed by 5 days of rest) have reversed epigenetic clock, reseted telomeres’ length over critical threshold and have also extended the life expectancy in a mouse model of accelerated ageing (Hutchinson Gilford Progeria Syndrome); these effects might also be applicable to supercentenarians [53,84].

Other proven effects of Yamanaka 4F overexpression in aged mice were: remarkable reduction in p53 binding protein 1 (53BP1) involved in the DNA damage; downregulation of the expression of p53-mediated age-related stress response genes and senescence-associated metalloproteases (MMP13), interleukins IL-6 and IL-8; restored level of methylated histones H3K9me3 and H4K20me3; restored skin histologic appearance and thickening; significant life-span increase. It has been hypothesized that Yamanaka 4F also reset the epigenetic clock of the stem cells, thus diminishing the stem cell pool exhaustion during ageing [45,91].

Moreover, transient overexpression for 4 days of combined transcription factors OSKMLN (OSKM+ LIN28+ NANOG) in adult human dermal fibroblasts and endothelial cells, as well as in progeroid mouse fibroblasts, has revealed significant rejuvenating effects and diminished cells age, due to the reduction of senescence biomarkers of DNA and nuclear envelope damage, dysregulation of histones, mitochondrial ROS production, SAβGAL and SASP. Moreover, OSKMLN has restored telomeres’ length, mitochondrial membrane potential, and increased sirtuin SIRT1 protein levels, extended mice lifespan, in the absence of teratomas formation [89,113].

**Figure 1:**
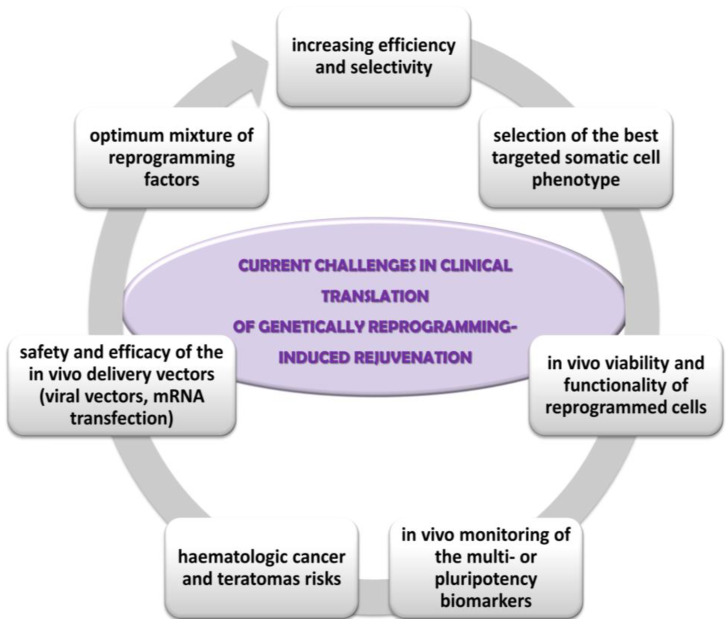
Current challenges in clinical translation of genetically reprogramming-induced rejuvenation.

However, since the great majority of successful rejuvenation studies have been performed on animal tissues without taking into account species-specific differences, there are some challenges for clinical translations of genetically reprogramming-induced rejuvenation, such as (Figure 1):

✓ the optimum mixture of reprogramming factors for each cell phenotype; low efficiency (about 25% of cells in culture being partially reprogrammed); lack of selective rejuvenation by reprogramming expression of genes which are not essential to ageing; lack of influence on some ageing hallmarks, such as mitochondrial DNA mutations, intracellular and extracellular metabolic aggregates [85,93];
✓ the selection of the somatic cell type targeted for reprogramming; fibroblasts are best candidates due to their proportion in the skin, supportive role, proliferative capacity, the implication of their contractile form (myofibroblasts) in the non-functional persistent scars [51];
✓ integration of the reprogrammed cells into the tissue physiology, especially in age diseases context; the persistence and the degree of the functionality of reprogrammed cells within the in vivo tissue microenvironment [87];
✓ insufficient optimization and monitoring of the reprogramming techniques; for instance, in vivo monitoring of the multi- or pluripotency biomarkers in order to minimize long-term tumorigenesis risk, the stability and viability of the rejuvenated cells in culture and in vivo, the rate of ageing of the younger phenotype cells in comparison with the normal, un-reprogrammed skin cells [91];
✓ carcinogenic risks: activation of oncogenes, point mutations in coding DNA associated with genomic instability and teratomas’ development; phenotypic mosaicism of partially reprogrammed stem cells can cause lineage bias, dysfunctional stem cell and higher haematologic cancer and teratomas risks [86,112];
✓ the safety and efficacy of the in vivo delivery vectors of the reprogramming transcriptional factors. For instance, viral vectors are associated with increased cancer risk because they can cause insertional mutagenesis, residual expression or re-activation of reprogramming factors, or might have broad organ-tropism [84,109,114]. Other safer delivery methods already tested are transient transfection with non-integrating viral vectors, mRNA transfection, or chemically induced reprogramming by small molecules and growth factors.

### 6.3. Chemically Reprogramming-Induced Skin Rejuvenation Strategies

Since multiple epigenetically regulated pathways have been involved in skin ageing, epigenetic skincare has become an emerging field in the design of small molecule active ingredients. Chemical-based epigenetic reprogramming comprises: inhibitors of DNA methyltransferases (DNMTs); inhibitors of the histone deacetylases (HDACs) or of the histone methylases; activators of sirtuin SIRT6; microRNAs (miRNAs) and miRNA’s inhibitors, as well as modulators of the long non-coding RNA (lncRNA) HOTAIR (HOX Transcript Antisense RNA), assisted or not by CRISPR-gene editing technology [57,89,115,116,117,118]. Currently, epigenetic drugs applied by chemical-based reprogramming approach avoid the use of oncogene *c-Myc* (one of the Yamanaka factors 4F) and have been validated in mice experiments and more than 20 have entered clinical trials [114].

#### 6.3.1. Small-Molecule Epigenetic Drugs: Main Inhibitors of HDAC and DNMT, and Activators of Sirtuin SIRT6

DNMT inhibitors, like 5-azacytidine, facilitate the reprogramming of human fibroblast into iPSCs, up-regulate their pluripotency gene expression and differentiation capacity towards other germ layers, confer human dermal fibroblasts a relaxed chromatin structure and short plasticity [89,93]. Histone methylation inhibitors also stimulate pluripotency-related gene expression. For instance, 3-deazaneplanocin A, an H3K27me3 and H4K20me3 inhibitor, activates *OCT4* during iPSC reprogramming [114]. HDAC inhibitors reverse the deacetylation of histone tails and trigger the specific gene expression related to epigenetic clock. They could be illustrated by the following examples. Rapamycin, that is also a senomorphic, might stimulate epigenetic reprogramming since it maintains the level of some histone biomarkers which are decreasing with age (i.e., H3R2me2, H3K27me3, H3K79me3, H4K20me2) [55,57]. Moreover, in a clinical trial, topically applied rapamycin has reduced p16^INK4A^ biomarker levels in ageing skin and DNAm age, after 6 months of therapy [57,62]. The isoflavone equol (formed by gut bacteria from soy isoflavone daidzein), topically applied for 8 weeks, decreases biomarkers of global DNA methylation and of telomere attrition, with visible improvements in skin roughness, texture, hydration and smoothness [88,112,117]. Sulforaphane from cauliflower, anacardic acid from *Anacardium occidentale* (cashew) nutshells, epigallocatechin-3-gallate (EGCG) from green tea, and palmitoyl-KVK-*L*-ascorbic acid conjugate, function as inhibitors of the DNA methyltransferase 1 (DNMT1, the most abundant enzyme responsible for DNA methylation) or of the enzyme HDAC, and they have stimulated the synthesis of procollagen, that is greatly involved in matrix repair, skin elasticity and strength [34,115].

Topical epigenetic skincare agents (e.g., equol, sulforaphane, retinoids, oligopeptides, etc.) are increasingly favoured for their safe, non-invasive and epigenome-targeted effects, although their clinical efficacy remains slow and moderate [117,118,119]. The main knowledge gaps in topical epigenetic modulators which must be further elucidated and validated are related to: (a) long-term safety; (b) optimal delivery systems; (c) bioavailability and penetration into deep dermal layers; (d) off-target epigenomic effects; (e) large randomized human trials [57,105,117,119,120].

Sirtuins are intensively investigated as HDAC modulators in epigenetic skin rejuvenating attempts [87]. Sirtuins are encoded by *Sir-2* gene, considered one of the longevity gene besides *p66shc*, *ink4a*, *FOXO* and *daf-2* genes, and have double catabolic activity both in deacetylation and ADP-ribosylation coupled to NAD^+^ hydrolysis. Among SIRT1-7 protein family, nuclear sirtuin SIRT6 is mostly studied for the prevention of immune skin dendritic cells’ (Langerhans cells) senescence [22]. Nuclear SIRT6 is classified as histone deacetylase whose activity significantly improves upon binding of fatty acids and whose major histone substrates comprise H3K9, H3K56 and H3K18; SIRT6 has also non-histone targets, such as: tumour protein p53, histone acetyltransferase GCN5, and tumour necrosis factor TNF-α. SIRT6 increases nicotinamide adenine dinucleotide (NAD) levels by its ADP-ribosyltransferase activity for mono-ADP-ribosylate nuclear proteins [51,89,114,121].

SIRT6 directly or indirectly control the promoters of the genes for main transcription factors and thus mediate crucial pathways for DNA repair, heterochromatin relaxation and compaction, antioxidant defence, epigenome maintenance, transcriptional regulation of metabolic processes, endothelial cell function and cellular ageing. Furthermore, SIRT6 overexpression efficiently activates and restores DNA repair of single-strand breaks and double-strand breaks, especially under oxidative stress conditions, thus contributing to longevity. In photoageing induced either by UV irradiation or genotoxic chemicals, SIRT6 facilitates the excision of bulky DNA adducts by nucleotide excision repair pathway [22,34,113,121]. SIRT6 is also significantly inversely correlated to human primary skin fibroblasts ageing; furthermore, SIRT6 overexpression expands by 27% the median lifespan in male mice and by 15% in female mice compared to wild type mice [22,113]. In addition, SIRT6 enhances: the integrity of telomeric chromatin and TRF2; epigenetic reprogramming efficiency in human dermal fibroblasts; preservation of repetitive sequences of heterochromatin organization; as well as maintenance of youthful epigenetic clock in correlation to lamin A/C (i.e., a nuclear scaffold protein involved in chromatin packaging and whose mutations were identified in Hutchison-Gilford progeria) [90,105,109,112,119].

Activators of SIRT6 as potential epigenetic skincare tested so far might be illustrated by: resveratrol and trans-polydatin (a more active glycosidic derivative of resveratrol isolated from *Polygonum cuspidatum*); MDL-800, an allosteric SIRT6 modulator promoting DNA repair and genomic stability [45,114]; cyanidin, a quercetin derivative that activates the in vitro SIRT6 deacetylase activity (by 55-fold increase); the alkaloid licorine (stimulates SIRT-mediated DNA repair in human fibroblasts); brown algae-isolated polysaccharide fucoidan (a strong in vitro activator of SIRT6) [22,45,53,121]; ergothioneine, that stimulates SIRT1 and SIRT6 expression, reduces mitochondrial oxidative stress and hyperglycemia; “SIRTfood”/sirtuin diet (food rich in sirtuins’ activators such as resveratrol) [113,121,122]. However, sirtuins’ uncontrolled overexpression can promote epithelial–mesenchymal dysplasia and therapeutic resistance, therefore further research on many experimental models, in a hormesis approach, is still needed [105,112,113,119].

Comparative insights into the most relevant emerging studies on small molecule epigenetic drugs as skin rejuvenation strategy, their principal outcomes in restoring epigenetic balance and their challenges towards clinical translation are summarized in Table 7. Small-molecule epigenetic drugs, such as inhibitors of HDAC and DNMT, or activators of sirtuin SIRT6, have proven to be a promising route to shift gene expression toward a youth-like state, in both systemic and topical application, thus enabling biological rejuvenating effects by reversing epigenetic clocks. However, they are currently limited by non-specific targeting, possible risk of epigenomic instability, systemic toxicity and dose sensitivity; they also lack robust clinical anti-ageing trials in human skin ageing, since most findings derive from animal models (e.g., remetinostat), cosmetic contexts or from skin cancer clinical interventions [84,105,119,120,123,124,125,126,127,128,129].

#### 6.3.2. MicroRNAs (miRNAs)-Based Modulators in Skin Rejuvenation

MicroRNAs (miRNAs) are gaining increasing interest as skin rejuvenating alternatives due to their potential of regulating the expression of some essential genes involved in ageing. MicroRNAs are small (~22 nucleotides) non-coding RNA molecules able to bind to messenger RNAs (mRNAs), either degrading them or inhibiting the translational process [116]. miRNA-based interventions, delivered via exosomes, nanoparticles or topical formulations, have high tissue specificity enabling skin-selective regulation of key ageing biomarkers of inflammation and senescence and also of collagen production; but they encounter great limitations and challenges related to the efficiency of transdermal delivery, stability, durability of effects, and off-target undesired regulatory reactions [118,130].

The great majority of miRNA-based therapeutics as skin rejuvenators is still in early preclinical studies as topical formulations with the potential to post-transcriptionally regulate the expression of genes associated with senescence. In early-stage clinical trials for skin rejuvenation there are tested some topical formulations and miRNA delivery systems, such as miR-21- and miR-146a-loaded nanoparticles [118,131]. The miRNAs and miRNA’s inhibitors which are mostly advanced in preclinical and clinical tests for skin anti-ageing purposes are summarized in Table 8:

✓ miR-29 family (miR-29a, miR-29b, miR-29c), which suppress the expression of MMPs within ECM, thus decreasing collagen degradation and maintaining skin’s collagen levels; they also upregulate the expression of collagen genes (*COL1A1*, *COL3A1*) [132];
✓ miR-146a, that interferes the NF-κB inflammatory pathway by targeting IL-1 receptor-associated kinase 1 (IRAK1) and TNF receptor-associated factor 6 (TRAF6); in mice models with UVB (ultraviolet B)-induced photoageing and inflammation, topical or injected miR-146a has reduced erythema, skin senescence markers and improved skin healing [133];
✓ inhibitors of miR-34a, which up-regulate the expression of the sirtuin 1 (SIRT1) gene involved in longevity and DNA repair; in vitro, inhibitors of miR-34a have stimulated proliferation of cultured fibroblasts and have diminished senescence biomarkers [134];
✓ inhibitors of miR-155, which have demonstrated the capacity to reduce the levels of inflammatory cytokines and pigmentation associated with chronic inflammation in aged human skin biopsies; inhibitors of miR-155 up-regulate the expression of the suppressor of cytokine signalling *SOCS1* gene; SOCS1 protein inhibits the JAK/STAT pathway (Associated Janus Kinases/Signal Transducers and Activators of Transcription) and therefore it has a crucial role as negative regulator of cytokine signalling in immune disorders, cancer and inflammation [132];
✓ miR-21, that targets inhibitors of skin regeneration and wound healing, such as phosphatase and tensin homologue (PTEN) and sprouty homologue 1 (SPRY1); miR-21 has stimulated fibroblasts’ division, collagen synthesis, keratinocyte migration and dermal matrix restoration, in in vivo murine wounds models [134];
✓ miR-200c, that has reduced the levels of oxidative stress and has regulated epithelial–mesenchymal transition (EMT) in in vitro studies, due to its capacity to target zinc finger transcription factors (E-box-binding homeobox 1 and 2, ZEB1 and ZEB2) [134].

Current hurdles in clinical progress of the miRNA-based skin anti-ageing therapies are related to:

✓ targeted and efficient delivery across stratum corneum into dermal layers, using as carriers exosomes, lipid nanoparticles, hydrogels [135];
✓ undesired gene silencing effects due to non-selective binding to genes responsible for essential cellular pathways [118,134];
✓ limited long-term safety data [136];
✓ in vivo rapid metabolic degradation by ribonucleases (RNases), requiring chemical modulations (e.g., 2′-*O*-methylation) [131,135];
✓ lack of data on inter-individual variability of miRNA efficacy due to age, race, hormonal status and previous skin diseases [116,118,132].

**Table 8:** The main microRNAs-based interventions preclinically and clinically tested as skin anti-ageing therapies (the arrow ↓ means decrease/inhibition; the arrow ↑ means increase/stimulation).

miRNA Approach	Regulation of Principal Target Gene(s)	Main Results	Stage	Ref.
miR-21	↓ *PTEN* ↓ *SPRY1*	↑ fibroblast proliferation and healing; ↑ collagen synthesis; ↑ keratinocyte migration and dermal matrix restoration	Preclinical, in vivo murine wounds models	[134]
		topical application of miR-21: improved skin elasticity and moisture; no major adverse effects	early clinical-phase I	[135]
miR-29a/b/c	↓ MMPs ↑ COL1A1	↓ ECM degradation; ↑ collagen production; ↑ dermal structure; ↓ wrinkles	Preclinical: in vitro/human dermal fibroblasts; animal models	[132]
miR-146a	NF-κB inflammatory pathway: ↓ IRAK1 ↓ TRAF6	topical or injected miR-146a: anti-inflammatory; ↓ UVB damage; ↓ erythema, skin senescence markers; ↑ skin healing	Preclinical: mice models of UVB-induced ageing	[133]
		topical application of miR-146a-loaded nanoparticles: ↑ skin elasticity and moisture; no major adverse reactions	early clinical—Phase I	[135]
inhibitors of miR-34a	↑ *SIRT1* gene	↑ fibroblasts’ proliferation and longevity; ↓ senescence biomarkers	Preclinical: in vitro fibroblasts cultures	[134]
inhibitors of miR-155	↑ *SOCS1*	↓ chronic inflammation; ↓ inflammatory cytokines; ↓ skin pigmentation	Preclinical: aged human skin samples	[132]
miR-200c	zinc finger E-box-binding homeobox *ZEB1*, *ZEB2*	↓ oxidative stress; ↑ epithelial–mesenchymal transition	Preclinical: in vitro	[94,130,134]

#### 6.3.3. Modulators of the Long Non-Coding RNA (lncRNA) HOTAIR in Skin Rejuvenation

HOTAIR (HOX Transcript Antisense RNA) is a long non-coding RNA (lncRNA) involved in chromatin remodelling, gene expression’s silencing by promoting histone H3K27 methylation, transcriptional regulation in specific genomic regions via interaction with EZH2, a catalytic subunit of the Polycomb Repressive Complex 2 (PRC2) [137,138]. The relevance of HOTAIR’s modulation for skin ageing derives from its involvement in dermal fibroblast senescence, wound healing impairment, skin cancer progression and epigenetic reprogramming resistance. Overexpression of HOTAIR is associated with fibroblast senescence, impaired ECM remodelling, fibrosis and pro-inflammatory signalling—features common to chronological ageing, photoageing and sclerotic skin conditions. High levels of HOTAIR contribute to UV-induced damage by promoting keratinocyte apoptosis and inflammatory cytokine expression [137,138,139]. However, in burn wound healing, HOTAIR supports regeneration by enhancing angiogenesis via miR126/Wnt/VEGF axis (Wnt/beta-catenin/Vascular Endothelial Growth Factor), indicating a context-dependent function [137,140]. Thus, inhibiting HOTAIR in acute injury may hinder recovery, highlighting the need for precise application and understanding of biological context and timing in therapeutic targeting [140].

HOTAIR is emerging as a multifunctional epigenetic regulator in the skin and its modulation, especially its inhibition in dermal fibroblasts and keratinocytes, may hold promise for anti-ageing skin strategies, particularly in photoaged and fibrotic skin [137,141]. Targeting HOTAIR by various means is illustrated in Table 9, besides of those featured by skin anti-ageing interventions assisted by gene-editing (CRISPR)-technology, and may restore youthful gene expression, reduce fibrosis and inflammatory signalling, enhance tissue regeneration pathways supporting skin rejuvenation [130,137,138,139,140,141,142,143].

**Figure 2:**
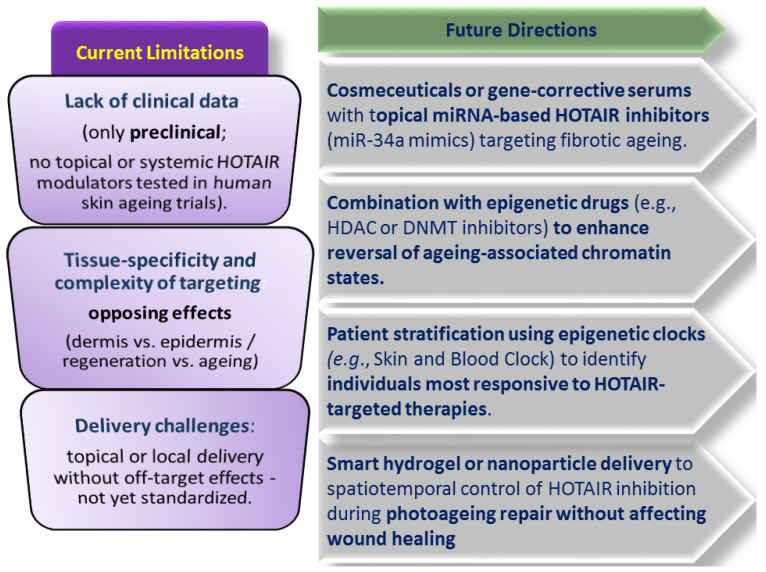
HOTAIR modulators in skin rejuvenation—current limitations and future research.

The role of HOTAIR modulators in skin rejuvenation strategies lies in their precision in targeting the age- and damage-associated epigenetic dysregulation in skin cells. Recent studies suggest that topical or systemic HOTAIR inhibitors might be integrated into epigenetic skin anti-ageing strategies, although most are still in preclinical (cellular or animal) stages [130]; human trials for topical or systemic HOTAIR modulators in skin ageing or rejuvenation are not yet reported. The clinical translation of the HOTAIR modulators will depend on topical delivery technologies, improved tissue specificity and the integration with epigenetic diagnostic tools. Current limitations and future research directions on integrating HOTAIR modulators into epigenetic skin rejuvenating strategies are illustrated in Figure 2.

#### 6.3.4. Gene Editing by CRISPR-Based Approaches in Skin Rejuvenation

Although not a therapy itself, Clustered Regularly Interspaced Short Palindromic Repeats (CRISPR)-based technology is part of gene therapy and it has demonstrated precision in genome editing and epigenetic remodelling, as well as rejuvenation potential. Gene-editing by CRISPR acts by direct modification of ageing-related genes or regulatory sequences. CRISPR-direct (CRISPRd) controls gene expression by precisely blocking specific transcription factor binding sites on DNA, without cutting/altering the DNA sequence. CRISPRd uses a deactivated Cas9 protein (dCas9) guided to a specific DNA site by an RNA molecule, where it physically prevents the binding of the transcription factors to that particular DNA site. For instance, ex vivo CRISPR/Cas9 is able to edit ageing-related genes in fibroblasts by targeting p^16INK4A^ or FOXO pathways. On the other hand, CRISPR-activation (CRISPRa) increases the expression of a specific gene by guiding an inactive CRISPR-Cas9 system (dCas9) fused with transcriptional activators to that gene’s promoter region, thus boosting its transcription and increasing the production of the gene’s encoded protein, also without altering the DNA sequence [144].

Although targeting transcriptional regulators (e.g., SOX5) via CRISPR have shown encouraging efficacy on correcting mutations and modulating ageing-related genes or rejuvenation pathways, the current data are largely preclinical, experimental as human skin applications, or are investigated in non-skin systems [144,145,146]. Furthermore, CRISPR/Cas9 faces critical ethical, regulatory, delivery and safety barriers before translation into dermatologic applications. Therefore, gene editing, although revolutionary, is still experimental in dermatology, requiring extensive safety and ethical evaluation [147]. Their combination with novel delivery methods like microneedles could further enhance gene editing specificity and safety, but need human clinical validation [144,145]. The main examples of the CRISPR-gene editing approaches in skin rejuvenation and a comparative analysis of this innovative approach are illustrated in Table 10 [127,144,145,146].

## 7. Skin Anti-Ageing Strategies Targeting Dysregulated Nutrient Sensing Pathways

Nutrient-sensing pathways comprising sirtuins, mTOR, IGF-1 and IGF-1 signalling (IIS) pathway, and AMPK, have a crucial role in mediating skin ageing [25,88]. Sirtuins, a family of signalling proteins essential in regulating metabolism, decline in aged subjects in parallel to reduced proliferation of dermal fibroblasts. Sirtuins have become especially activated during calorie/diet restriction and by the water-soluble form of vitamin B3—NAD. Applied topically, NAD has induced the expression of sirtuins and increased mitochondrial energetic chain, clinically reflected in improvement of skin ECM barrier and skin pigmentation [88,122].

Resveratrol at concentrations < 10 μM acts as antioxidant and senomorphic, preventing cellular senescence and diminishing SASP, either by activation of sirtuin SIRT1, PI3K-Akt signalling pathway and telomerase, or by inhibition of NF-κB pathway, depending on the cell type. At concentrations > 25 μM, resveratrol acts as pro-oxidant and stimulator of senescence and/or apoptotic cell death in many cell types. This biphasic effect seems to be dependent not only on concentrations, but also on administration schedule, cell types, animals’ sex, genetic profile and diet composition [55,122,148]. Multiple sirtuin-activating compounds, especially SIRT1 activators, have proven superior stability and bioavailability than resveratrol, such as the compound SRT1720 that has 1000-fold increased potency, as well as the compound SRT2104 that is well tolerated and has good bioavailability in clinical trials for age-related diseases [58,149]. Topical creams with 0.01% capsaicin, applied for over 7 days, have stimulated the production of dermal IGF-1 through the activation of sensory neurons and the most noticeable clinical sign reported was the significant improvement of cheek skin elasticity [14,44]. Rapamycin, an FDA-approved drug that inhibits the mTOR complex, has improved both the appearance and histology of skin in numerous participants [23,62,63,122].

## 8. Skin Anti-Ageing Strategies Targeting Mitochondrial Dysfunction

As skin ageing proceeds, the increasing accumulation of mitDNA mutations reduces mitochondrial integrity with significant mitochondrial fragmentation and clustering, impairs the oxidative phosphorylation in human skin fibroblasts and keratinocytes, and triggers metabolic conversion from oxidative phosphorylation to anaerobic glycolysis [24,150]. For instance, in chronological age, there are reported: an increased deletion rate of the 4977 base pair mitDNA, a marked decrease in mitochondrial membrane potential, a decrease in coenzyme Q10 (CoQ10) and also an increase in the ROS levels in fibroblasts. In photoageing skin, studies have revealed a 10-fold increased accumulation of mitDNA mutations in skin fibroblasts than in protected skin, increased levels of collagen-degrading metalloproteases and down-regulation of genes involved in collagen biosynthesis [29,151]. Antioxidants, such as: resveratrol, quercetin, vitamin C and E, curcumin, CoQ10, melatonin, epicatechins, phytoestrogens, epitalon (a synthetic tetrapeptide composed of Ala-Glu-Asp-Gly), or synthetic antioxidants nanoparticles, have been tested to alleviate mitochondrial oxidative stress [112,150]. Topical CoQ10 application have improved by 44% the mitochondrial membrane potential and skin smoothness by reducing wrinkles [31,148]. Elamipretide, a mitochondrial-targeted antioxidant, might also improve chronic wound healing [24,151].

Anti-ageing nanomedicines based on nanozymes with artificial enzymes mimicking superoxide dismutase, peroxidase, glutathione peroxidase, catalase, NADH oxidase or cytochrome c oxidase, have been developed by various structural modulations of active centres (i.e., metal oxide, noble metal, metal–organic-framework, single-atom catalysts) in order to regulate the mitochondrial respiratory chain, to generate NAD, to alleviate oxidative stress, as well as to improve structural stability, functional catalytic diversity, and to be easily and cost-effectively mass-produced [152,153]. For instance, Pd-based single-atom nanozyme Pd@PPy/GO mimicking peroxidase and glutathione peroxidase have been endowed with precise coordination metal-support solid bonds and significantly higher quantum size interactions and catalytic activity, thus making it a promising mitochondrial anti-ageing nanozyme. Moreover, C60(C(COOH)2)3 superoxide dismutase like nanozyme, as well as Au, gallic acid and isoflavone based nanozymes, delivered inside mitochondria have diminished age-related oxidative stress in mice skin, while ZnO-nanozymes decreased global DNA methyltransferase as age-related epigenetic alteration in cultured fibroblasts [153,154]. However, nanozymes are currently limited by some disadvantages in comparison to natural enzymes, such as lower substrate selectivity and specificity, potential systemic toxicity, the risk of cytotoxic byproducts, restrained simultaneous catalytic activities [152].

## 9. Comparative Analysis of Genomic/Epigenomic-Based vs. Classical Skin Rejuvenation Treatments

In Table 11, there is a comparative analysis among the newer (epi)genomic skin anti-ageing strategies and classical ones, from various perspectives: efficacy, safety, mechanism of action, evidence level in preclinical and clinical data, price range, and current limitations. (Epi)genomic-based skin anti-ageing strategies target fundamental molecular ageing processes and mechanisms and therefore they could enable more profound and long-lasting rejuvenation effects, than symptom-focused classical approaches. A comparative analysis on the efficacy and safety of the (epi)genomic-based skin anti-ageing strategies tested so far is quite difficult to be clearly assessed, because there is a great heterogeneity in studies’ design and standardization concerning the intervention type, transdermal and targeted delivery methods, mechanistic and clinical endpoints and outcome measurement tools. However, tretinoin remains the gold standard with well-documented gene modulation effects, but side effects limit patient adherence, while newer genomic-based strategies are very promising, but they still lack validation through large-scale and long-term follow-up randomized controlled clinical trials [22,53,59,61,62,63,77,78,89,90,118,119,135,136,142,144,145,147,155,156,157,158,159,160,161,162,163].

## 10. Other Clinically Tested Anti-Ageing Strategies

### 10.1. Anti-Ageing Approach of Inflammageing

The concept of inflammageing proposed by Franceschi in 2000 encompasses chronic age-related inflammation and is connected to altered intercellular communication, fibroblasts senescence and SASP (MMP, IL-1β, IL-6) in aged skin [18,148]. The process of chronic inflammation and itching in ageing skin is mediated by the following factors: systemic increase in pro-inflammatory cytokines, coupled to diminished number and responsivity of the Langerhans cells (immune dendritic cells) in aged epidermis; increased prevalence of senescent keratinocytes and of melanocytes characterized by pro-inflammatory IL-1α secretory capacity; alteration of the epidermal permeability barrier due to elevated pH; augmented activity of kallikreins involved in the degradation of epidermal structure. In addition, dysfunctional immune T cells, pro-inflammatory T helper (Th)17 phenotype and supression of local adaptative immune mechanisms might also mediate chronic inflammation in aged skin [21,22,33]. In order to mitigate inflammageing, topical use of nanoemulsion formulation of a tocotrienol-rich fraction have shown promising efficacy in reducing UV-induced chronic inflammatory signs [1,11]. Moreover, melatonin acts as anti-inflammatory agent, inhibitor of premature photo-induced senescence in melanocytes and of p53 activity, stimulator of antioxidant enzymes and DNA damage repair, as well as anti-apoptotic agent [58,163,164].

### 10.2. Anti-Ageing Strategies Targeting Altered (Macro)autophagy and Intercellular Communication

(Macro)Autophagy is a catabolic pathway contributing to the eukaryotic cells survival under various stress factors (DNA damage, nutrient deprivation, hypoxia, excessive reactive oxygen radicals, intracellular pathogens), because it removes cellular debris, damaged organelles, toxic and misfolded proteins, thus maintaining cellular homeostasis, angiogenesis, melanogenesis and increasing the skin resistance to external stress [1,22,39,90]. Macroautophagy is continuously active in the rapidly renewing epidermal epithelium during the cornification of keratinocytes, and has proven to be crucial in long-lasting skin cells (i.e., Merkel cells, melanocytes, sweat gland secretory, sebaceous glands) [14,21].

As autophagy activators topically tested in clinical trials can be cited: Aquatide™, a heptasodium hexacarboxymethyl dipeptide, that proved a statistically significant enhancement in skin elasticity and significant decrease in carbonylated proteins in the stratum corneum, after 4–8 weeks of application; MflCas, a phytocosmetic topical preparation with extracts from *Myrothamnus flabellifolia* leaf and *Coffea arabica* seed, that has reduced hyperpigmentation, wrinkle area and volume, and has also ameliorated skin homogeneity and luminosity, after 56 days of use [58,86,90]; A^+^skin serum^®^ (a growth factor-based skincare serum obtained from human fibroblast-conditioned media), eventually prepared with magnetized saline water, that has enhanced facial and neck skin biophysical parameters, as well as having significantly reduced the overall hyperpigmentation, coarse and fine wrinkles, and sagging. Moreover, A^+^skin serum^®^ and topically applied exosomes from various sources (bovine milk, human placental mesenchymal stem cells) have restored connexins and enhanced moisture retention, collagen biosynthesis and skin repair mechanisms [148,151].

### 10.3. Anti-Ageing Strategies Targeting Recovery of Skin Proteostasis

Both in chronological and photo-induced aged skin, loss of skin proteostasis or altered skin proteome is mainly caused by: glycation and carbonylation of the proteins with the accumulation of AGEs and oxidized proteins, as well as altered protease secretion in the dermal fibroblasts; declining in autophagy; increased MMP secretion; down-regulation of the hyaluronic acid synthases in aged epidermal stem cells, dermal fibroblasts, melanocytes, keratinocytes; increased cross-links between collagen and elastin fibres, protein misfolding and degradation. Carnosine, resveratrol, extracts from *Akebia quinata* and bacteria *Arthrobacter agilis* have proven active in protection of skin cells proteostasis, recovering skin firmness and elasticity [23,58,149].

### 10.4. Anti-Ageing Strategies Targeting Skin Dysbiosis

Aged skin is marked by a greater microbial diversity with the following variations: a significant increase in cheek and forehead *Corynebacterium* (especially *Corynebacterium kroppenstedtii* and *Corynebacterium amycolatum*), and scalp *Acinetobacter*; a decrease in cheeks, forehead and forearms *Cutibacterium*; as well as high prevalence of *Firmicutes* in acne-predisposed adults, no matter their age. Targeting skin dysbiosis focuses on topical prebiotics, probiotics, bacteriocins (i.e., antimicrobial peptides produced by bacteria) which might reduce the prevalence of pro-inflammatory bacteria (e.g., *Staphylococcus aureus*) within skin microbiome [109,164].

## 11. Conclusions and Future Perspectives

Progresses in epigenomics and genomics underlying the accelerated ageing pathophysiology have deciphered and enabled new perspectives on the complexity, relationships and intricate pathways which should be addressed. Thus, various strategies specifically designed to target more accurately the main hallmarks of skin photoageing have already achieved promising results either in animal studies or even in clinical trials. In the future, some current limitations especially regarding methodological optimization, toxicological risks, or ethical issues, require further investigation and validation on larger cohorts, in order to translate all these achievements into approved strategies [28,97,163].

The design of future clinical trials would have to:

✓ prioritize the integration of epigenomic clock biomarkers (DNAm, Horvath’s, GrimAge) as well as the multi-omics platforms (epi/genomics-transcriptomics-metabolomics-proteomics) in order to quantitatively assess epigenetic and biological age reversal;
✓ expand the correlation among epigenomic and genomic biomarkers, histologic assessment and clinical outcomes;
✓ elucidate the relationship between the dose and administration period and the (epi)genomic response;
✓ define the long-term safety implications of genomic modulation;
✓ reinforce the clinical validation of efficacy and toxicological profile by enrollment of larger and well-defined cohorts, increase the statistical power (at least over 100 participants), by application of placebo control and blinding (especially with half-face designs) in longer-term RCTs, by prolongation of the follow-up (at least 1 year) period for the evaluation of the desired and unwanted effects’ persistence and possible regression [44,63,118,119,135,136,144,147,155].

Formulation challenges of the products containing epigenetic and genetic bioactive molecules would have to address physico-chemical and in vivo stability, skin penetration enhancement, as well as targeted delivery within deep dermal layers and bioavailability of genomic/ epigenomic agents by using nanotechnology, advanced carrier systems, optimized targeted genomics-informed delivery vehicles (e.g., smart hydrogels, nanoparticles, exosomes, niosomes, nanosomes delivery, etc.) and synergistic potential of combination therapies between classical and genomic/epigenomic strategies.

**Figure 3:**
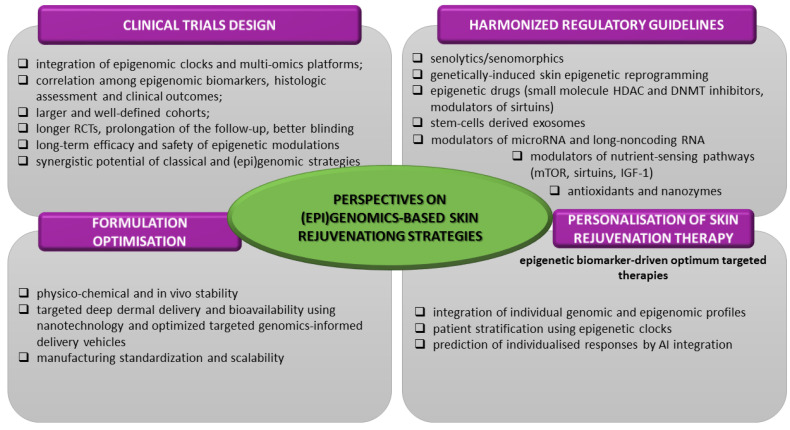
Future perspectives in (epi)genomic-based skin rejuvenation.

Future research in this field should also be dedicated to continuous development and implementation of harmonized regulatory guidelines for approval of senescence-targeting therapies for skin ageing: senolytics/senomorphics, epigenetic reprogramming, epigenetic drugs, miRNAs, modulators of nutrient-sensing pathways, and of skin microbiota [28,44,104,119,144]. New perspectives should also focus on personalization of the skin anti-ageing strategies by integration of the individual genomic and epigenomic profiles to customize/tailor skin rejuvenation therapies [119,144,147]. Future research perspectives in (epi)genomic-based skin rejuvenation are depicted in Figure 3.

Regarding translational potential and market accessibility of these emerging (epi)genomic-based skin antiageing strategies from niche, premium procedures, to widespreadly adopted ones, manufacturing feasibility and economic scalability remain the major determinants. In this respect, their cost-modelling analysis would enable an estimative stratification of them into near-term, mid-term, and long-term adoption strategies.

Among the strategies with the highest near-term adoption (≈5–10 years) likelihood could be mentioned: senotherapeutics, epigenetic modulators, and nano-delivery systems, based on their strong balance of biological rationale and clinical evidence, lower manufacturing cost, better scalability, and regulatory tractability [59,165]. Senotherapeutics show high/rapid translational and near future accessibility rate due to cost-effective production and supporting evidence in skin models, since many of them are repurposed active small molecules with already proved clinical and safety profile (e.g., topical rapamycin, peptide senotherapeutics in ex vivo skins), although their specificity for skin senescent cells remains to be further demonstrated in clinical studies without impairing wound healing. Senotherapeutics offer one of the best cost–benefit ratios, with cheap synthetic molecules (costing at scale USD 1–5 per dose; USD 5–50 per doses at administration), while peptide senomorphics cost more (USD 5–60) but still remain much more affordable than other biologics [50,165,166].

The experts consider epigenetic modulators (small molecules, topical epigenetic agents, topical NAD^+^/sirtuin-modulators, NAD^+^ boosters, HDAC inhibitors) translationally tractable in near-term/ early mainstream integration, since they are already inexpensive and scalable, and their widespread adoption is primarily gated by clinical evidence for meaningful skin rejuvenation, not by manufacturing constraints. Epigenetic small-molecule modulators are low-cost because of simple standard synthetic chemistry and mass production, typically under USD 1 manufacturing cost and administration costs below <USD 20 per topical-equivalent dose (some targeted chromatin-modifier drugs are more expensive) [51,59].

The adoption of nanotechnologies (nanocarriers for topical delivery, lipid and polymeric NP) is also expected to be rapid and broad, because they are industrially scalable (especially lipid NP) and they have already clearly proven long-term safety profiles. In general, nanotechnologies (e.g., nanoformulated antioxidants, peptides) scale efficiently and fall into mid-range pricing, depending on NP type and encapsulation [167]. NP are characterized by low–moderate overall cost barrier, high manufacturing scalability, and cost-efficiency; since the industrial-scale NP synthesis is mature and scalable, NP have modest costs per clinical dose, depending on route (topical NP $1–15/dose; injectable forms $20–150/dose) [168].

Moderate adoption is expected for *microRNA* therapeutics because they are more economically tractable, have moderate costs due to scalable chemical oligonucleotide synthesis (estimate administration costs USD 50–500 per dose equivalents), mature industrial NP formulation processes, and lower quality control (QC) burden. The major bottlenecks in their wide clinical adoption are target validation and optimized delivery specificity, stability and clear regulatory pathways [116,118,132,169].

Despite its promising, high biological and translational potential, the exosomes’ therapy is appreciated by experts as a mid-term, slower/wider-risk adoption strategy, because of the regulatory complexity, manufacturing and standardization gaps (due to complex upstream production, stringent and costly QC requirements), and uncertain clinical evidence for many anti-ageing interventions. Exosomes have still the highest cost among the regenerative approaches, therefore they will likely expand first in well-funded esthetic or clinical trial settings. The EV’s main GMP manufacturing cost drivers are expensive bioreactor culture, purification, potency testing, and donor-source dependence, as well as modest production yields; all these are reflected in very high costs estimated to USD 8000–20,000 per manufacturing batch and ~USD 150–550 per dose. In addition, the administration/clinical-level pricing is much higher (estimated to USD 300–1500 per clinical dose), since it carries a significant markup above raw production cost, including regulatory risk, marketing, clinician time, facility overhead, and perceived value (anti-ageing demand); however, these are estimative costs which vary a lot among peer-reviewed studies, industry reports and experts assumptions. Standardization of many GMP manufacturing pathways, especially of the producer cell lines and continuous purification pipelines, are compulsory to reduce costs and to convert EV from premium procedures into mass-market products. Therefore, EV’s market accessibility and adoption will greatly depend on industrial process innovation than on scientific validation alone [170,171,172].

Epigenetic reprogramming, while scientifically compelling due to epigenetic clock reversal and huge skin rejuvenation potential, remains at the frontier of biomedical research as a long-term and high-impact strategy, whose translation path towards mass adoption would require several decades. Partial epigenetic reprogramming is an extremely high-cost strategy, by far the most expensive (over USD 10,000–100,000 per vector batch) and technically demanding due to GMP-grade viral or non-viral vector engineering, major safety constraints and intensive off-target risk monitoring, and regulatory/ethical hurdles. Its adoption will be slow, restricted, and likely confined to specialized medical centres for the foreseeable future [25,38,84,114].

The above data sustaining the stratification of the emerging (epi)genomic-based skin rejuvenation strategies according to their widespread accessibility and market adoption are synthesized in Table 12.

**Figure 4:**
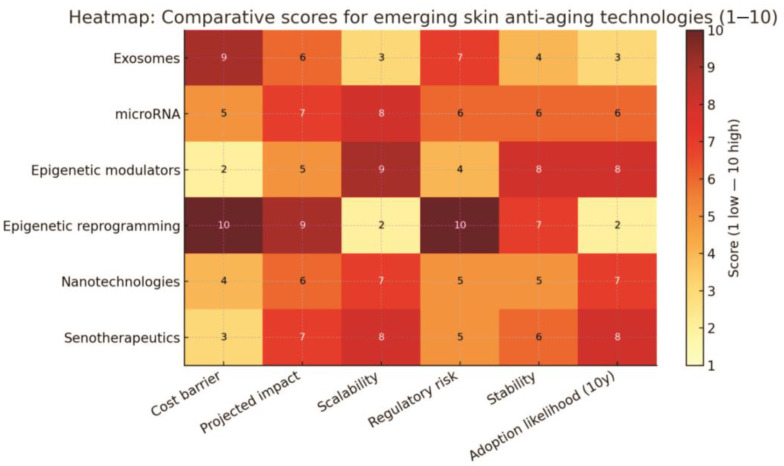
Heatmap of estimative widespread adoption of the emerging (epi)genomic-based skin rejuvenation strategies.

The accessibility and widespread adoption of the emerging (epi)genomic-based skin rejuvenation strategies is also illustrated in Figure 4 as a heatmap in which to each strategy is assigned an estimative comparative score by integrating expert-opinions and evidence-based reports regarding their production complexity, clinical progress, regulatory status, sample stability, known and predicted costs from scalable manufacturing and administration prices.

## Figures and Tables

**Table 1:** Types of age(ing).

Type of Ageing	Definition	Limitations	Reference (Ref.)
Intrinsic (chronologic/calendar) (skin) age	Genetically programmed, time-dependent skin changes seen without external damage.	Lacks measureable molecular markers; occurs slowly.	[3]
Biological (phenotypic) age	Reflects how well a person’s body (or skin) is functioning relative to peers, using functional or molecular data.	Requires biomarker panels; may lack tissue specificity.	
Epigenetic age (DNAm age, CpG clock)	Age estimated from deoxyribonucleic acid DNA methylation (DNAm) patterns at CpG sites, correlates with cellular ageing.	Needs lab-based testing; clocks vary in accuracy and clonal specificity.	[4,5]
Skin age (dermal age)	Biological or epigenetic age of skin tissue specifically, often with skin-optimized clocks.	Dependent on validated skin-specific models; requires biopsies or swabs.	[6]
Photoageing (UV-ageing, photodamage)	Premature ageing of skin caused by chronic ultraviolet exposure.	Primarily assessed clinically or histologically, not quantified by molecular clocks.	[3,7]
Pace of ageing (ageing rate, velocity)	Rate at which biological ageing progresses over time, often via longitudinal DNAm measures.	Not absolute age; indirect for skin unless validated in skin tissues.	[7]
Senescence (cellular ageing markers)	State of irreversible cell-cycle arrest, often with Senescence-Associated Secretory Phenotype (SASP) and markers like p16^INK4A^.	Requires invasive sampling and is qualitative rather than quantitative.	[8]
Inflammageing (age-related inflammation)	Chronic low-grade systemic or skin inflammation marked by cytokines (e.g., interleukin IL-6, C reactive protein CRP, tumour necrotic factor TNF-α).	Non-specific; fluctuates with environmental or health variables.	[9]

**Table 2:** Comparative insights into intrinsic aged versus photo-aged skin.

Feature	Chronologically Aged Skin	Photoaged Skin	Ref.
Mechanistic pathways	Progressive mitochondrial decline, oxidative stress, modest MMP activity	UV-induced oxidative stress → MAPK/AP-1 and NF-κB pathways’ activation, MMP upregulation, transforming growth factor TGF-β suppression	[15,16,17]
Clinical signs	Fine lines, thinning, dryness, reduced elasticity, generally even skin tone	Deep wrinkles, rough texture, pigment spots, telangiectasias, laxity	
Histological changes	Thinned epidermis and dermis, decreased fibroblasts	Epidermal hyperplasia, solar elastosis, collagen fragmentation, vascular dilation	
ECM, collagen and elastin modifications	Slow ECM degradation via baseline MMP; gradual loss of dermal collagen; mild elastin fibre disorganization developing with age	Rapid collagen breakdown by UV-induced MMP-1/-3/-9; suppressed collagen synthesis; sever elastin fibre disorganization	
Inflammation	Low-grade systemic inflammageing	Chronic localized inflammation: elevated IL-6, TNF-α, ROS in photoexposed skin	
DNA damage and Senescence	Gradual telomere shortening, modest senescence	UV-triggered DNA mutations (e.g., p53), telomere attrition, accelerated senescence	
Mitochondrial dysfunction	Progressive oxidative damage derived from cells’ metabolism	Acute UV-induced mitochondrial injury causing ROS burst and oxidative stress	

**Table 3:** Comparative insights on various epigenetic and non-epigenetic clocks/biomarkers relevant to skin rejuvenation strategies’ evaluation.

Clock	Mechanism	Clinical Advantages (Skin and Regenerative Medicine)	Clinical Limitations	Ref.
EPIGENETIC CLOCKS				
Horvath clock (1st generation)	DNAm at 353 CpGs across tissues	Universal tissue clock; baseline age marker for cell-based therapies	Not skin-optimized; slow responsiveness to short-term interventions	[4]
Horvath Skin and Blood clock (2nd generation)	DNAm at 391 CpGs (skin/fibroblast-derived)	Highly accurate for skin ageing; ideal for assessing rejuvenating interventions	Requires full methylome data; limited systemic insights	[6]
GrimAge (2nd generation)	DNAm proxies for plasma proteins and smoking	Robust mortality predictor; sensitive to systemic drugs (e.g., metformin)	Not specific to skin; limited in localized skin interventions	[37]
PhenoAge (2nd generation)	blood DNA methylation patterns linked to clinical ageing-related biomarkers (e.g., CRP, albumin, white blood cells WBC, glucose)	Well validated in predicting: biological age, systemic ageing, mortality risk, and chronic disease prediction; accessible in blood-based anti-ageing research.	Not designed or validated on skin tissue; inaccurate for skin ageing assessment	[5]
DunedinPACE (3rd generation)	DNAm-based rate-of-ageing (longitudinal)	Measures short-term ageing pace; suitable for anti-ageing trials	Trained on blood; indirect for skin anti-ageing interventions	[7]
miRNA Skin clock (3rd generation)	Skin-specific miRNA transcriptomics	Non-invasive; emerging tool for cosmetic/pharma intervention studies	Not fully validated; lower accuracy (MAE ~8–10.9 years)	[27]
Rayan Skin Clock	Tissue-specific epigenetic clock trained on human facial epidermal and dermal fibroblast methylation data, spanning ages 18–90.	Highly accurate and specifically optimized for skin ageing, especially for facial skin; sensitive to esthetic anti-ageing treatments; enables objective and accurate quantification of skin biological age changes after esthetic procedures (like laser resurfacing); detects reversible reprogramming signatures unique to skin; superior to general clocks (e.g., Horvath, PhenoAge) for skin applications.	Requires skin biopsy or high-quality epidermal samples; not yet commercially available for clinical use (mainly applied in research settings); complex methylation analysis pipeline; not validated for darker skin tones or other body areas	[26]
NON-EPIGENETIC CLOCKS/MARKERS				
Telomeres’ length	Telomeric DNA attrition	Historically used; available assays	Poor specificity for skin ageing; weak intervention tracking	[42]
Senescence biomarkers	p16^INK4A^, SAβGAL, SASP cytokines	Mechanistic; reflects rejuvenation via senolytics, retinoids, etc.	Requires tissue samples; not systemically quantifiable	[8]
Inflammatory biomarkers	IL-6, TNF-α, CRP (inflammageing)	Reflects chronic skin inflammation; useful in photodamage assessment	Non-specific; transient variations; overlaps with immune response	[9]

**Table 5:** Knowledge gaps and future research directions in senotherapeutics as skin rejuvenating strategy.

Knowledge Gaps	Future Research Directions	Ref.
Safety and off-target effects ✓ currently short-term effects—days to weeks; ✓ systemic senolytics (like navitoclax/ABT-263) can induce apoptosis in non-senescent cells due to their action on BCL-2 family proteins; ✓ peptides/ botanical extracts still demand rigorous safety profiling.	Long-term safety evaluation ✓ at least 6 months—1 year; ✓ off-target effects (apoptosis) on other non-senescent, proliferating or quiescent cell-types by influencing similar critical pathways; ✓ long-term effects on skin homeostasis, microbiome or skin barrier integrity; ✓ immunosenescence; ✓ neoplastic risk	[10,12,19,44,50,51,59,82,83]
Formulation ✓ still poor dermal penetration; ✓ invasive delivery methods limiting cosmetic use	Transdermal targeted delivery ✓ selective targeted delivery to senescent dermal fibroblasts or epidermal keratinocytes; ✓ advanced or combined delivery systems: nanotechnology, microneedles, ultrasound	
Translation from lab to human Most senotherapeutics evaluated in murine models or ex vivo human skin, which cannot fully replicate the complexity of human skin ageing and interindividual variability. Ongoing and closed clinical trials—limited enrolment and diversity: most trials *n* = 22–74 healthy middle-aged women.	Clinical trials on long-term safety and efficacy Clinical trials design: ✓ larger studies (*n* > 100), broader demographic data (older adults); ✓ longer durations, long-term effects in diverse groups; ✓ primary end-points: safety, optimal dosage and long-term benefits on human skin; ✓ better control or stratification of lifestyle factors; ✓ expanded inclusion criteria (photoaged skin, pigmentation disorders, compromised barriers, etc.)	
Individualisation of the antiageing senotherapeutics still greatly unaddressed	Biomarker-driven personalization ✓ new robust and non-invasive biomarkers (e.g., skin autofluorescence, microRNA panels) for better monitoring of antiageing responsivity; ✓ profiling patient-specific senescence biomarkers (p16^INK4A^ or methylome clocks); ✓ integration with artificial intelligence (AI) and machine learning (ML) models to predict patient-specific responses to various senotherapeutics	
Combination approaches currently, few tested	Synergistic combinations ✓ sequential regimens and adjunct energy-based modalities; ✓ senotherapeutics with microneedling, laser resurfacing or platelet-rich plasma; ✓ senotherapeutics with retinoids or antioxidants	

**Table 6:** Clinical trials on stem-cells derived exosomes (EV) as skin anti-ageing strategy (the arrow ↓ means decrease; the arrow ↑ means increase).

Approach	Trial Design	Main Outcomes	Limitations	Ref.
Stem-cell EV + microneedling	12-week prospective randomized split-face RCT (*n* = 28 women aged 43–66), 3 sessions over 12 weeks: Arm 1 = 12.5% ADSC-derived EV + microneedling vs Arm 2 = saline + microneedling	Significant improvement on treated side vs. control of wrinkles, elasticity, hydration, pigmentation; higher Global esthetic improvement scale (GAIS) scores on treated side vs. control ↑ collagen histologically; transient erythema/petechiae; no serious adverse effects (AEs)	Small cohort; short follow-up; split-face design (within-subject placebo); possible crossover effects; lack of proper placebo	[94,105]
Stem-cell EV + fractional CO_2_ laser: acne scars	12 weeks, RCT (*n* = 25): acne scars; Adipose-derived stem cell exosomes (ADSC-exo) + laser vs. laser + control gel	Greater acne-scarring improvement; milder erythema and shorter downtime vs. control gel	Small size; scar-focused; short follow-up; limited generalizability	[97,100]
Umbilical cord stem cell- and adipose-derived stem cell-conditioned medium and exosomes, via topical or microneedling	*n* ≈ 22–30 participants aged 18–69 years; 3–10 weeks, often adjunct to laser or microneedling	Anti-photoageing effects, modulating signalling pathways involved in skin damage and promoting collagen synthesis; ↑ dermal density, ↑ collagen/elastin genes’ expression; improved wrinkles, hydration, pigmentation; minimal side effects	The adjunctive therapies make hard to isolate effects; short follow-up; small sample sizes	[105,106,108]
Topical stem-cell conditioned medium + laser resurfacing	Meta-analysis of 5 RCTs	Significant reduction in wrinkles, pigmentation, pore size; improved overall skin condition	Heterogeneity; varied protocols; multiple small RCTs	[102,105]
Topical ADSC-exosomes for brightening	8 week double-blind RCT (*n* = 21 women): topical ADSC-EV vs. placebo; split-face	Significant melanin reduction at 4 weeks; effect sustained up to 8 weeks; no AEs	Small size samples; limited topical penetration; modest clinical brightness effect	[98,105,107]
MSC-derived exosomes ointment	6 weeks, *n* = 56 healthy adults, 40–85 years; topical 3D imaging study; split-face histopathologic evaluation	Histopathologic evaluation: ↓ wrinkles, redness, melanin; ↑ luminosity, even tone; safe and well tolerated	Single-arm, no control; non-randomized; short duration; moderate sample size	[95,96,101,103]
ADSC-EV injection	Preclinical: UVB-rat photoaged skin	↓ Epidermal thickening; ↑ dermal thickness; ↑ collagen I; ↓ collagen III; ↓ MMP-1/3 expression	No clinical data; single dose	[105]
Exosome skincare + defensins serum	Multi-centre double-blind vehicle-controlled RCT of defensin-containing regimen	Demonstrates clinical and histopathologic benefit	Limited detailed evaluation	[104]
Exosomes from genetically engineered stem cells	Phase I/II clinical trials for skin rejuvenation	Delivery of growth factors, miRNAs, epigenetic modulators; skin texture improvement; increased collagen synthesis	Manufacturing standardization; scalability	[94]
Human platelet extract (HPE) exosome product	60 participants (40–80 years), dorsum of hands; 56 participants facial, with 20 biopsies	HPE matched vitamin C in improving texture/tone; well tolerated; ↑ collagen and elastin histologically	Small biopsy sub-cohort; unclear skin-type distribution; no long-term follow-up	[95]
MSC growth factor serums	Split-face RCT, 3 months; compare MSC’s growth factor serums vs fibroblast’s growth factor serums; 20 participants with moderate–severe photodamage	Both serums significantly improved wrinkles without difference; well tolerated	Small sample size; unclear penetrance mechanisms; half-face design may confuse effects	[99,101]
Intradermal cell therapy: RCS-01 Skin Rejuvenation RepliCel	Randomized, double-blind, placebo-controlled phase I clinical trial (completed). safety-focused Phase I; small cohort; intradermal injection of collagen-expressing hair-follicle fibroblasts	Non-bulbar dermal sheath (NBDS) cells are prolific producers of tissue building proteins especially type I collagen (5× that of dermal fibroblasts). NDBS cells have been shown to promote in vivo tissue collagen and ECM regeneration; up to 2× increase in gene expression of collagen-related biomarkers; healthier, younger-looking skin after a single injection; no serious AE	Small cohort; not powered for efficacy; only surrogate biomarkers of gene-expression	[108]

**Table 7:** Comparative insights into the main emerging clinical and preclinical studies on small molecule epigenetic agents as skin strategies.

Small Molecule Epigenetic Agents	Study Design	Main Outcomes	Limitations/Challenges	Ref.
Histone deacetylase (HDAC) inhibitors: topical remetinostat	Phase II open-label, single-arm clinical trial: topical remetinostat gel in skin cancer (basal cell carcinoma)	Complete clinical and pathological resolution of tumours; epigenetic modulation of skin cells; good dermal penetration; potential for skin anti-ageing use	Cancer context, no direct anti-ageing endpoints; small cohort; off-target effects possible	[120,123,124]
Topical sirtuin activators (e.g., resveratrol)	Clinical cosmetic studies; some early-phase trials ongoing	Improved skin hydration, elasticity and antioxidant activity	Mostly cosmetic; small samples size; limited direct epigenetic biomarker data; poor bioavailability	[45,109,114,125]
Topical DNA methyltransferase (DNMT) inhibitors	Early preclinical stages	Potential reversal of aberrant methylation in aged skin	Lack of clinical trials; unclear safety in humans	[105]
Restorative Skin complex [RSC] and TriHex™ RSC + Tripeptide-1 + Hexapeptide-12 (Alastin Skin Care/Galderma company)	22 subjects, 12 week facial applications	Proof of efficacy on in vitro fibroblast and keratinocyte; significant upregulation of the *Klotho* gene and related *FGF23, FGFR1* and *FOXO3B* longevity genes; significant telomere stabilization by shortening reduction over control (for RSC at 4weeks and for TriHex™ at 6 weeks); positive ECM activation: stimulation of collagen, fibrillin, CD44 and elastin	Relatively small cohort; non-competitive design; limited validation of in vivo mechanism	[107]
Hydroxyurea (epigenetic modulator)	Phase I/II trials in photoaged skin	Improved skin texture and reduced pigmentation	Cytotoxicity risks; precise mechanisms in ageing unclear	[119,126]
Modulators of ECM synthesis targeting TGF-β signalling	Clinical trials for skin fibrosis and ageing-related skin laxity	Increased collagen; improved skin firmness	Side effects in systemic application	[127]
Telomerase activators (e.g., TA-65)	Small-scale human trials; cosmetic application	Potential anti-ageing effects via telomere elongation	Safety concerns; limited large-scale studies	[120,128]
BET (Bromodomain and extra-terminal domain proteins) inhibitor (e.g., JQ1)	Preclinical phase only	Modulator of gene transcription by disrupting the interaction of BRD4 with acetylated histones	Potential toxicity; no clinical data	[119,129]

**Table 9:** Modulators of HOTAIR with anti-ageing relevance (the arrow ↓ means decrease/inhibition; the arrow ↑ means increase/stimulation).

Type of HOTAIR’s Modulators	Examples and Mechanism of Action on HOTAIR	Main Preclinical Results	Limitations	Ref.
miRNA mimic	miR-34a mimic inhibits HOTAIR; ↓ GLI2 * via Notch signalling → ↓ fibrotic gene expression	↓ α-SMA *; ↓ type I collagen; ↓ COL1A1; ↓ fibroblast proliferation and migration in systemic sclerosis dermal fibroblasts	Only in vitro, on disease-specific model from systemic sclerosis rather than on photoaged or normal skin	[137]
	miR-141, miR-203: direct binding to HOTAIR RNA and post-transcriptional repression			
HOTAIR- siRNA knockdown	direct silencing of HOTAIR in HaCaT human keratinocytes line; ↓ PKR → ↓ NF-κB and PI3K/AKT pathways’ activation	In cell-targeted treatments, anti-photo-ageing effects: ↓ UVB-induced apoptosis; ↓ IL-6, TNF-α cytokine release; improved keratinocyte survival	Immortalized cell line only (only epidermal model); lacks fibroblast or in vivo support	[139,141]
HOTAIR- short hairpin RNA (shRNA **) knockdown	In vivo HOTAIR knockdown: → ↑ miR-126 → activates Wnt/VEGF signalling pathway	Accelerated angiogenesis; faster wound healing in mice burn model	Context-dependent: inhibition may impair regeneration in normal skin	[140]
Natural compound regulators	Genistein, EGCG (green tea polyphenol): modulate lncRNA networks and epigenetic enzymes; Curcumin suppresses HOTAIR expression indirectly via epigenetic remodelling	HOTAIR downregulation shown in cancer models; potential extrapolation for reducing inflammatory lncRNA expression in skin ageing	Not tested in skin models directly; unclear bioavailability in topical delivery	[142]
Epigenetic drugs (indirect)	HDAC or DNMT inhibitors (e.g., resveratrol, curcumin) inhibit HOTAIR expression; GSK126, an EZH2 inhibitor, blocks HOTAIR binding partner PRC2 → epigenetic reactivation of suppressed anti-ageing genes	Reduced fibrotic gene expression in other models; potential to reverse HOTAIR-EZH2 repression in aged skin	No direct skin ageing studies; possible off-target effects	[138]
Antisense oligonucleotides (ASOs ***)	LNA-GapmeRs, siRNAs ****: RNA degradation or inhibition of lncRNA–protein interaction	Functional knockdown; studies in other ageing contexts	No direct skin ageing studies	[143]
CRISPRd/Cas9 tools	Block HOTAIR promoter activity	Transcriptional silencing; studies focus on other tissues	No evidence yet on skin ageing models	[130]

(*) GLI2: a zinc finger transcription factor, essential in regulating gene expression involved in cell survival, proliferation, and differentiation; PKR: Protein kinase R; α-SMA: alpha-Smooth muscle actin; PIK3: Phosphoinositide 3-kinase (cell survival signalling pathway); AKT: Protein kinase B (involved in survival and growth signalling); PRC2: Polycomb Repressive Complex 2; (**) Short hairpin RNA (shRNA): a molecule used in gene silencing, specifically within RNA interference; it is a synthetic RNA sequence designed to form a hairpin structure within a cell, which is then processed into siRNA, ultimately leading to the silencing of a targeted gene; (***) Antisense oligonucleotides (ASOs) are short, synthetic single-stranded RNA or DNA molecules, highly specific and customizable through chemical modulations, designed to bind to specific messenger RNA sequences, thereby regulating protein expression; (****) Locked nucleic acid (LNA): a modified RNA analogue, with conformational rigidity, high specificity, and enhanced stability.

**Table 10:** Comparative insights into main CRISPR- gene editing approaches in skin rejuvenation.

Gene Editing/CRISPR-Assisted Interventions	Study Design	Main Outcomes	Limitations/Challenges	Ref.
CRISPRa SOX5 activation in senescent cells	Preclinical gene therapy in senescent cells and aged mouse cartilage	Epigenetic remodelling via gene activation: genome-wide CRISPR activation screening in senescent human precursor cells + aged mice gene therapy. SOX5 activation remodels epigenome, reduces senescence; gene therapy improved aged cartilage	Joint ageing model; reduced pro-ageing gene expression and inflammation; no skin data yet	[144]
Microneedle-delivered CRISPR-Cas9	Preclinical animal studies delivering CRISPR gene editors into skin via dissolvable microneedle arrays	Efficient local gene editing in skin tissue; minimal invasiveness	Translation to humans pending: untested efficacy, safety, off-target effects in humans	[145]
CRISPR/Cas9 correction of *Krt9* mutation	Mice monogenic epidermolytic palmoplantar keratoderma- (EPPK)-like disease	Lentiviral CRISPR/Cas9 targeting *Krt9* mutation in mice epidermis: approx. 14.6% reduction in mutant *Krt9*; improved epidermal differentiation	Small effect	[127]
Optogenetic CRISPR	Mice skin	Demontrated spatially precision in gene editing	Proof-of-concept; only animal model	[146]

**Table 11:** Comparative insights between classical and (epi)genomic skin anti-ageing strategies.

Insight	Classical Treatments	Genomic/Epigenomic-Based Treatments	Ref.
Efficacy	Tretinoin (all-trans retinoic acid): clinically proven to reduce fine lines, hyperpigmentation, and skin roughness over ~16 weeks of daily topical use; Peptide-based cosmeceuticals (palmitoyl pentapeptide-4, other collagen-modulating peptides): moderate evidence of stimulated collagen synthesis; Intense pulsed light IPL: moderate improvement in skin pigmentation and tone; Laser combinations (e.g., ablative CO_2_ with intense pulsed light IPL): over 40% improvement in skin texture and firmness; Platelet-rich plasma (PRP) + microneedling: moderate increase in collagen production after 2–3 sessions; Chemical peels (glycolic acid, TCA, salicylic acid): moderate to high efficacy for fine lines, pigmentation, texture; Botulinum toxin injections: high efficacy for expression lines; Dermal fillers (hyaluronic acid, calcium hydroxylapatite): immediate visible improvement; duration varies (lasting months to years depending on filler).	Senolytic/Senomorphic agents (dasatinib + quercetin, fisetin, etc.): promising preclinical and early clinical data for reversing senescence biomarkers; Topical rapamycin (an mTOR pathway inhibitor): reduces both the expression of p16^INK4A^ and DNA methylation age; stimulates elastin; clinical improvement in dermal thickness; Epigenetic bioactives (e.g., sulforaphane, equol): reduce LINE-1 methylation and global methylation; improve skin barrier function, hydration and smoothness; MicroRNA(miRNA) modulators: topical application of miR-21- and miR-146a-loaded nanoparticles improved skin elasticity and moisture; no major adverse effects (phase I clinical trials); Topical DNA repair enzymes: moderate reduction in photoageing and UV damage; CRISPR-based gene modulation: theoretical potential to reverse age-related gene expression patterns, but not yet in clinical use.	[22,53,59,61,62,63,77,78,89,90,118,119,135,136,142,144,145,147,155,156,157,158,159,160,161,162,163]
Safety/toxicity	Tretinoin: common adverse effects include mild skin irritation, peeling, and erythema, which are generally transient (typically subside after 2–4 weeks); Peptide-based cosmeceuticals (palmitoyl pentapeptide-4, other collagen-modulating peptides): well tolerated; Topicals: well tolerated; occasional transient erythema; IPL: mild redness, swelling; rare pigment changes; Laser resurfacing: possible adverse events include scarring, dyspigmentation, and prolonged erythema; side effects are reduced with combination therapy; Microneedle/laser combinations: mild procedural side effects (erythema, edema, petechiae); PRP + microneedling: generally safe; infection or scarring if improperly performed; Intradermal cell transfer: no serious adverse effects in phase I studies; Chemical peels (glycolic acid, TCA, salicylic acid): mild irritation, redness; scarring or prolonged erythema after stronger peels; risk of hyper-/hypo-pigmentation; Botulinum toxin injections: generally safe; occasional bruising or ptosis; Dermal fillers (hyaluronic acid, calcium hydroxylapatite): mild bruising, swelling; rare vascular occlusion.	Senolytic/Senomorphic agents (dasatinib + quercetin, fisetin); generally short-term safety data; long-term safety unknown; Topical rapamycin: demonstrated minimal systemic absorption and adverse effects in studies up to 8 weeks (well tolerated short-term); Epigenetic bioactives: typically well tolerated; limited data on long-term safety in large-scale populations; MicroRNA (miRNA) modulators: still unknown; Topical DNA repair enzymes: well-tolerated; CRISPR technologies: still experimental in dermatology; potential off-target effects; ethical concerns.	
Downtime (post-therapy recovery from visible side effects or symptoms)	Tretinoin: minimal downtime; mild redness and dryness may occur for a few days; Peptide-based cosmeceuticals (palmitoyl pentapeptide-4, other collagen-modulating peptides): not applicable; IPL: 1–3 days redness/swelling; Laser resurfacing (e.g., fractional CO_2_ laser): redness, swelling and peeling for 1–2 weeks; full healing up to 6 months depending on aggressiveness; Microneedling: 1–3 days of mild erythema; Chemical peels (glycolic acid, TCA, salicylic acid): variable from none to 7–14 days depending on peel concentration and depth; Botulinum toxin injections: minimal; 1–2 days mild swelling; Dermal fillers (hyaluronic acid, calcium hydroxylapatite): minimal; 1–7 days swelling/bruising.	Senolytic/Senomorphic agents: unknown; Epigenetic topical therapies (e.g., rapamycin, sulforaphane): no downtime observed; MicroRNA (miRNA) modulators: still unknown; Topical DNA repair enzymes: none; Gene-editing treatments (theoretical): delivery should be non-invasive or systemic; expected minimal surface recovery time but currently not clinically available.	
Mechanism	Tretinoin: enhances dermal collagen synthesis and epidermal turnover; Peptide-based cosmeceuticals (palmitoyl pentapeptide-4): stimulate collagen synthesis; Laser therapy: induces controlled dermal injury thus promoting neocollagenesis and elastin production; IPL: broad-spectrum light for pigmentation, vascular lesions, and collagen stimulation; PRP + microneedling: skin injury + growth factor stimulation; triggers fibroblast activation and growth factor release; Chemical peels (glycolic acid, TCA, salicylic acid): controlled exfoliation; Botulinum toxin injections: blocks neuromuscular signalling to reduce dynamic wrinkles; Dermal fillers (hyaluronic acid, calcium hydroxylapatite): restores volume, smooths static wrinkles; Growth factor serums: replenish endogenous signaling, but epidermal penetration and transport remain unclear.	Senolytic/Senomorphic agents (dasatinib + quercetin, fisetin): clear senescent cells; Rapamycin: inhibits mechanistic target of rapamycin (mTOR), reducing cellular senescence markers like p16^Ink4A^; Epigenetic regulators: modulate DNA methylation and histone acetylation to restore youthful gene expression; MicroRNA (miRNA) modulators: preclinical evidence for modulating the expression of essential genes involved in ageing pathways (e.g., SIRT1, SOCS1, MMP, COL1A1, ZEB1 and ZEB2, NF-κB pathway, etc.); Topical DNA repair enzymes: T4 endonuclease V creams enhance repair of UV-induced DNA damage; CRISPR-based gene editing interventions: edit or silence ageing-related genes.	
Cost	Tretinoin: relatively inexpensive (USD 100– USD 300/ approx. EUR 90– EUR 270 per session); Peptide-based cosmeceuticals (palmitoyl pentapeptide-4): USD 50– USD 150 (EUR 45– EUR 135) per product; IPL: USD 300– USD 700 (EUR 270–EUR 630) per session; Laser resurfacing: high cost (~USD 1500–USD 6500; or approx. EUR 1350–EUR 5850 per session); Microneedling + PRP: ~ USD 500–USD 1500/approx. EUR 450–EUR 1350 per session; Chemical peels (glycolic acid, TCA, salicylic acid): USD 100– USD 300 (EUR 90–EUR 270) per session; Botulinum toxin injections: USD 300– USD 600 (EUR 270–EUR 540) per treatment; Dermal fillers (hyaluronic acid, calcium hydroxylapatite): USD 500– USD 1200 (EUR 450–EUR 1080) per syringe.	Senolytic/Senomorphic agents: unknown; Topical rapamycin: experimental; costs unclear, compounding costs; Nutrigenomic cosmeceuticals (e.g., sulforaphane creams): USD 50–$150 (EUR 45–EUR 135) per product; MicroRNA (miRNA) modulators: unknown / no approved products; Topical DNA repair enzymes: USD 100–$200 (EUR 90–EUR 180) per product; CRISPR and RNA therapies: currently confined to research settings; potential high cost due to advanced biotech manufacturing.	
Evidence level and Regulatory approval	Supported by multiple randomized controlled trials and decades of clinical data; FDA-approved indications for tretinoin, DNA repair enzymes (for acne and photodamage); FDA-cleared for: IPL devices for skin rejuvenation; fractional lasers for resurfacing; Many registered as cosmetic product categories without strict regulation.	Histological and gene-level evidence confirm ECM remodelling (↑collagen/elastin); Intradermal and microneedle-assisted therapies tend to show stronger tissue-level changes; All modalities report improvements in wrinkles, elasticity, hydration, pigmentation within 6–12 weeks; Limited to small cohort studies (e.g., topical rapamycin pilot studies, human subjects, 6 months). Preclinical or early-phase (I) human trials for sulforaphane and other epigenetic bioactives, some miRNA; CRISPR and RNA therapies not yet tested in dermatology patients.	
Limitations/knowledge gaps	Tretinoin: requires long-term use; skin irritation—common side effect; photosensitivity; Peptide-based cosmeceuticals (palmitoyl pentapeptide-4): limited robust clinical evidence; effects mild and gradual; IPL: multiple sessions needed; not effective on deep wrinkles; Laser resurfacing: expensive; requires skilled operator; potential prolonged recovery; Microneedling + PRP: variable results; risk of infection if not performed correctly; Chemical peels (glycolic acid, TCA, salicylic acid): not suitable for all skin types; risk of hyper-/hypo-pigmentation; Botulinum toxin injections: temporary effect (~3–6 months); repeated treatments needed; Dermal fillers (hyaluronic acid, calcium hydroxylapatite): risk of vascular complications; temporary results; cost varies; Growth factor serums: unclear epidermal penetration and transport.	Senolytic/Senomorphic agents: early-stage research; human data limited; delivery methods challenging Topical rapamycin: limited clinical data; long-term safety and efficacy not fully established Topical epigenetic cosmeceuticals: mostly preclinical data; unclear clinical efficacy; unclear optimal dosing and formulation Topical DNA repair enzymes: limited efficacy on deeper ageing signs (wrinkles); requires regular use; variable efficacy MicroRNA (miRNA) modulators: still in experimental phase; safety unknown Overall clinical trials design: small sample sizes (mostly n < 60); limited statistical power, control and blinding (lack proper placebo; possible crossover effects in split-face designs; unclear long-term durability (duration (mostly ≤12 weeks)	

**Table 12:** Estimative stratification of emerging (epi)genomic-based skin rejuvenation strategies according to their widespread accessibility and adoption.

Emerging (Epi)Genomic-Based Skin Rejuvenation Strategy	Cost-Modelling Prediction and Estimative Costs	Scalability	Regulatory/ QC Challenges	Ref.
NEAR-TERM ADOPTION STRATEGIES				
Senotherapeutics	Very favourable cost–benefit ratio; small molecules are very cheap to produce; peptides cost more but still affordable. Overall cost barrier: low–moderate. Manufacturing cost: low–moderate. Administration cost: USD 5–50/dose.	High	Specificity, systemic effects, peptide purity, formulation	[50,59,165,166]
Epigenetic modulators	Lowest cost barrier; simple chemistry allows mass-scale affordability. Manufacturing cost: low Administration cost: <USD 20/dose	Very high	Specificity, long-term impact	[51,59]
Nanotechnologies	Cost-efficient: industrial-scale NP synthesis is mature. Overall cost barrier: low–moderate. Scalable NP have modest costs per clinical dose, depending on route. Manufacturing cost: moderate. $1–15 (topical), USD 20–150 (injectable). Administration cost: USD 10–200/dose	High	Regulatory clarity, long-term biodistribution and safety, NP size control	[167,168]
MID-TERM ADOPTION STRATEGIES				
microRNA modulators	Mid-cost class; price range significantly reduced by scalable synthesis. Manufacturing cost: moderate for oligo synthesis (USD 500–5k/batch). Administration cost: USD 50–500/dose	High	Delivery, off-target effects, clear regulatory pathway	[116,118,132,169]
Exosomes (EVs)	Overall highest cost barrier; driven by GMP donor-source variability and QC load. Manufacturing cost: very high, USD 8–20k/batch (due to GMP cell culture, isolation, purification, and QC); USD 150–550/ per clinical dose (current mid-scale); scale-up needed to lower cost Administration cost: USD 300–1500/session	Low–moderate	Manufacturing standardization, safety profile	[170,171,172]
LONG-TERM ADOPTION STRATEGY				
Epigenetic reprogramming	Highest technological and economic barrier. Overall cost barrier: very high due to vector engineering and intensive safety needs. Manufacturing cost: extremely high (>USD 10–100 k/batch; USD 5000–40,000/dose). Administration cost: USD 2k–10k+	Very low	GMP vector manufacturing, safety monitoring, cancer risk	[25,38,84,114]

## Data Availability

No new data were created or analyzed in this study.

## Abbreviations

The following abbreviations are used in this manuscript:

ACS	Autologous conditioned serum
AD-MSCs	Adipose-derived mesenchymal stem cells
ADSC-exo	Adipose-derived stem cell exosomes
AE	Adverse event
AGEs	Advanced glycation end products
AI	Artificial intelligence
AKT	Protein kinase B (involved in survival and growth signalling)
AMPK	AMP-activated protein kinase (energy-sensing enzyme)
AMSCs	amniotic membrane stem cells
ASO	Antisense oligonucleotides
ATM	Ataxia telangiectasia mutated (kinase activated by DNA damage)
Bcl-2	B-Cell lymphoma 2 (anti-apoptotic protein)
BCS	Blood cell secretome
BET	Bromodomain and extra-terminal domain proteins
circRNA	circular RNAs
COL8A1	collagen type VIII alpha 1 chain
CoQ10	coenzyme Q10
CRISPR	Clustered regularly interspaced short palindromic repeats
CRISPRa	CRISPR activation (gene upregulation method, without altering the DNA sequence)
CRISPRd	CRISPR-direct
CRP	C reactive protein
D + Q	Dasatinib + quercetin (a common senolytic drug combination)
DDR	DNA damage repair
DNA	Deoxyribonucleic acid
DNAm	DNA methylation (age)
DNMT1	DNA methyltransferase 1
DRI	*D*-Retro-inverso (peptide configuration for therapeutic stability)
ECM	Extracellular matrix
EGCG	Epigallocatechin-3-gallate
EGFR	Epidermal growth factor receptor
ELN	Elastin
EMT	Epithelial–mesenchymal transition
EPPK	Epidermolytic palmoplantar keratoderma
EV	Exosomes/extracellular vesicles
EWAS	Epigenome-wide association study
4F	Yamanaka factors or OSKM factors: OCT4, SOX2, KLF4 and c-Myc
FOXO4	Forkhead box O4 (transcription factor involved in senescence survival signalling)
FOXO4-DRI	FOXO4-*D*-retro-inverso-isoform peptide
GAIS	Global esthetic improvement scale
GLS1	Glutaminase-1
GPNMB	Glycoprotein non-metastatic melanoma protein B (senescence surface marker)
GWAS	Genome-wide association study
HaCaT	Human keratinocyte cell line
HACS	Human adipose tissue-derived exosome-containing solution
HDAC	Histone deacetylase
HDACi	Histone deacetylase inhibitor
HIF1α	Hypoxia-inducible factor 1-alpha (transcription factor in low oxygen)
HOTAIR	HOX transcript antisense RNA
HPE	Human platelet extract
hUC-MSCs	Human umbilical cord mesenchymal stem cells
HUVEC	Human umbilical vein endothelial cells
i.p.	Intraperitoneal
i.v.	Intravenous
IGF-1	Insulin-like growth factor 1
IIS	IGF-1 signalling pathway
IKK	IκB Kinase (activates NF-κB)
IL-6, IL-8	Interleukin-6, interleukin-8 (pro-inflammatory cytokines, part of SASP)
IPL	Intense pulsed light
i-PRF	Injectable platelet-rich fibrin
iPSCs	Tissue-induced pluripotent stem cells
IRAK1	Interleukin 1 receptor-associated kinase 1
JAK/STAT pathway	Associated Janus kinases/ Signal transducers and activators of transcription
Krt9	Keratin 9 gene
lncRNA	Long non-coding RNAs
LOX	Lysyl oxidase
MAE	Mean absolute error (related to skin epigenetic clocks)
meQTL	Epigenome-wide methylation quantitative loci
miRNA	microRNAs
ML	Machine learning
MMPs	Matrix metalloproteases
MSCs	Mesenchymal stem cells
mTOR	Mechanistic target of rapamycin (a central regulator of cell growth and metabolism)
NAD	Nicotinamide adenine dinucleotide
NBDS	Non-bulbar dermal sheath cells
ncRNA	Non-coding RNA
NF-κB	Nuclear factor kappa-light-chain-enhancer of activated B cells (inflammation pathway)
OGG1	8-Oxoguanine glycosylase photolyase
OSKM factors	Yamanaka transcription factors or 4F: OCT4, SOX2, KLF4 and c-Myc
OSKMLN factors	Transcription factors OSKM + LIN28 + NANOG
p16^Ink4A^	Cyclin-dependent kinase Inhibitor 2A (senescence marker)
p53-pS15	p53-Serine15 phosphorylation
PDGF	Platelet-derived growth factor
PI3K	Phosphoinositide 3-kinase (cell survival signalling pathway)
PKR	Protein kinase R
PPARα	Peroxisome proliferator-activated receptor alpha (nuclear receptor)
PRC2	Polycomb Repressive Complex 2
PRF	Platelet-rich fibrin
PRP	Platelet-rich plasma
PTEN	Phosphatase and TENsin (tumour suppressor gene)
QC	Quality control
RCT	Randomized controlled trial
Ref	Reference
RNA	Ribonucleic acid
RNase	Ribonuclease
ROS	Reactive oxygen species
SAHF	Senescence-associated heterochromatin foci
SASP	Senescence-associated secretory phenotype (pro-inflammatory profile of senescent cells)
SAβGal	Senescence-associated β-galactosidase (marker enzyme for senescent cells)
shRNA	Short hairpin RNA
SIRT	Sirtuin
SOCS	Suppressor of cytokine signalling
α--SMA	alpha-Smooth muscle actin
SPRY1	Sprouty homologue 1
TCA	Trichloracetic acid
TGF	Transforming growth factor
TNF	Tumour necrotic factor
TRAF6	TNF receptor associated factor 6 (inflammatory signalling mediator)
TRF2	Telomeric repeat binding factor 2
UVB	Ultraviolet B
VEGF	Vascular endothelial growth factor
WBC	White blood cells
